# Supplementary material for: Association between social isolation and depression onset among older adults: a cross-national longitudinal study in England and Japan
Source: BMJ Open. 2021 Mar 18;11(3):e045834. doi: 10.1136/bmjopen-2020-045834 (PMC7978252; doi:10.1136/bmjopen-2020-045834)
Supplement: Supplementary data [file bmjopen-2020-045834supp001.pdf]

Supplementary Table 1. Association between social isolation and depression onset, by complete case analysis

		ELSA		JAGES	
		Crude OR (95% CI)	Adjusted OR (95% CI)	Crude OR (95% CI)	Adjusted OR (95% CI)
Social Isolation Index score	0	1.00 (reference)	1.00 (reference)	1.00 (reference)	1.00 (reference)
	1	1.92** (1.19–3.10)	1.56† (0.94–2.60)	1.14 (0.94–1.39)	1.11 (0.87–1.41)
	2	2.15** (1.28–3.62)	1.71† (0.98–2.99)	1.32** (1.09–1.60)	1.20 (0.95–1.51)
	3	3.19*** (1.73–5.90)	2.50** (1.27–4.90)	1.57*** (1.30–1.90)	1.30* (1.03–1.64)
	≥ 4	3.85** (1.46–10.18)	5.17** (1.83–14.66)	2.26*** (1.83–2.79)	1.47** (1.13–1.91)
		p for trend < 0.001	*p* for trend < 0.001	*p* for trend < 0.001	*p* for trend < 0.001
Age (years)	65–69		1.00 (reference)		1.00 (reference)
	70–74		1.37 (0.83–2.25)		1.16** (1.05–1.30)
	75–79		1.24 (0.71–2.14)		1.31*** (1.16–1.48)
	80–84		1.66† (0.92–2.99)		1.60*** (1.37–1.88)
	≥ 85		0.97 (0.45–2.09)		1.79*** (1.39–2.30)
Gender	Male		1.00 (reference)		1.00 (reference)
	Female		1.58* (1.07–2.34)		1.00 (0.90–1.11)
Educational attainment (years)	< 15		1.00 (reference)		1.00 (reference)
	16–18		1.05 (0.68–1.60)		0.81*** (0.74–0.89)
	≥ 19		1.30 (0.70–2.41)		0.68*** (0.60–0.78)
Household equivalised income	1st quintile (lowest)		1.00 (reference)		1.00 (reference)
	2nd quintile		0.70 (0.40–0.21)		0.95 (0.83–1.09)
	3rd quintile		1.11 (0.66–1.87)		0.76*** (0.67–0.87)
	4th quintile		0.82 (0.45–1.50)		0.67*** (0.57–0.79)
	5th quintile (highest)		0.84 (0.42–1.68)		0.65*** (0.56–0.76)

Cancer	No	1.00 (reference)	1.00 (reference)
	Yes	0.95 (0.32–2.81)	1.16 (0.93–1.43)
Heart disease	No	1.00 (reference)	1.00 (reference)
	Yes	1.13 (0.67–1.92)	1.20 (1.05–1.37)
Stroke	No	1.00 (reference)	1.00 (reference)
	Yes	1.3 (0.56–3.02)	1.18 (0.80–1.73)
Self-rated health	Good	1.00 (reference)	1.00 (reference)
	Poor	1.71* (1.13–2.59)	1.63*** (1.44–1.85)
CES-D score at baseline		1.90*** (1.60–2.25)	
GDS 8 score at baseline			1.92*** (1.86–1.99)
Smoking	Never/past	1.00 (reference)	1.00 (reference)
	Current	0.76 (0.76–1.56)	1.29*** (1.12–1.48)
Drinking	Never/past	1.00 (reference)	1.00 (reference)
	Current	1.41 (0.81 – 2.44)	1.02 (0.93 – 1.13)

*, $p < 0.05$; **, $p < 0.01$; ***, $p < 0.001$; †, $p < 0.1$

ELSA, English Longitudinal Study of Ageing; JAGES, Japan Gerontological Evaluation Study; CES-D 8, eight items from the Center for Epidemiologic Studies Depression Scale; CI, confidence interval; GDS, Geriatric Depression Scale; OR, odds ratio

Supplementary Table 2. Association between sub-components of social isolation and depression onset, by complete case analysis

		ELSA		JAGES	
		Crude OR (95% CI)	Adjusted OR (95% CI)	Crude OR (95% CI)	Adjusted OR (95% CI)
Social Isolation Index sub-components (ref; none)					
Unmarried or living alone		1.91*** (1.34–2.72)	1.27 (0.84–1.93)	1.33*** (1.18–1.49)	1.02 (0.88–1.19)
Poor interaction with children		1.08 (0.68–1.74)	1.51 (0.91–2.50)	1.01 (0.98–1.10)	1.13* (1.02–1.25)
Poor interaction with relatives		1.25 (0.83–1.90)	1.33 (0.85–2.08)	1.05 (0.97–1.13)	0.98 (0.90–1.08)
Poor interaction with friends		1.19 (0.78–1.80)	1.21 (0.78–1.87)	1.23 (1.14–1.33)	1.11* (1.01–1.22)
No social participation		1.54* (1.08–2.19)	1.46† (0.98–2.16)	1.70 (1.56–1.86)	1.22*** (1.09–1.36)
Age (years)	65–69		1.00 (reference)		1.00 (reference)
	70–74		1.37 (0.83–2.25)		1.17** (1.05–1.30)
	75–79		1.25 (0.72–2.17)		1.31*** (1.16–1.49)
	80–84		1.68† (0.92–3.04)		1.60*** (1.36–1.88)
	≥ 85		1.00 (0.46–2.21)		1.78*** (1.38–2.30)
Gender	Male		1.00 (reference)		1.00 (reference)
	Female		1.59* (1.07–2.37)		1.00 (0.89–1.11)
Educational attainment (years)	< 15		1.00 (reference)		1.00 (reference)
	10–12		1.04 (0.67–1.60)		0.81*** (0.74–0.90)
	≥ 19		1.31 (0.70–2.46)		0.69*** (0.61–0.78)
Household equivalised income	1st quintile (lowest)		1.00 (reference)		1.00 (reference)
	2nd quintile		0.71 (0.41–1.23)		0.95 (0.83–1.09)
	3rd quintile		1.13 (0.67–1.93)		0.76*** (0.67–0.86)
	4th quintile		0.83 (0.45–1.52)		0.67*** (0.57–0.79)
	5th quintile (highest)		0.85 (0.42–1.71)		0.66*** (0.57–0.76)
Cancer	No		1.00 (reference)		1.00 (reference)
	Yes		0.95 (0.32–2.81)		1.15 (0.93–1.43)

Heart disease	No	1.00 (reference)	1.00 (reference)
	Yes	1.16 (0.69–1.96)	1.20** (1.05–1.37)
Stroke	No	1.00 (reference)	1.00 (reference)
	Yes	1.28 (0.55–2.99)	1.18 (0.80–1.73)
Self-rated health	Good	1.00 (reference)	1.00 (reference)
	Poor	1.71* (1.13–2.58)	1.62*** (1.43–1.83)
CES-D 8 score at baseline		1.89*** (1.59–2.25)	1.00 (reference)
GDS score at baseline			1.92*** (1.85–1.99)
Smoking	Never/past	1.00 (reference)	1.00 (reference)
	Current	0.74 (0.36–1.53)	1.29*** (1.13–1.48)
Drinking	Never/past	1.00 (reference)	1.00 (reference)
	Current	1.39 (0.80–2.40)	1.03 (0.93–1.14)

*, $p < 0.05$; **, $p < 0.01$; ***, $p < 0.001$; †, $p < 0.1$

ELSA, English Longitudinal Study of Ageing; JAGES, Japan Gerontological Evaluation Study; CES-D 8, eight items from the Center for Epidemiologic Studies Depression Scale; CI, confidence interval; GDS, Geriatric Depression Scale; OR, odds ratio
