# Supplementary material for: Association between social isolation and depression onset among older adults: a cross-national longitudinal study in England and Japan
Source: BMJ Open. 2021 Mar 18;11(3):e045834. doi: 10.1136/bmjopen-2020-045834 (PMC7978252; doi:10.1136/bmjopen-2020-045834)
Supplement: Author's manuscript [file bmjopen-2020-045834.draft_revisions.pdf]

BMJ Open is committed to open peer review. As part of this commitment we make the peer review history of every article we publish publicly available.

When an article is published we post the peer reviewers' comments and the authors' responses online. We also post the versions of the paper that were used during peer review. These are the versions that the peer review comments apply to.

The versions of the paper that follow are the versions that were submitted during the peer review process. They are not the versions of record or the final published versions. They should not be cited or distributed as the published version of this manuscript.

BMJ Open is an open access journal and the full, final, typeset and author-corrected version of record of the manuscript is available on our site with no access controls, subscription charges or pay-per-view fees (http://bmjopen.bmj.com).

If you have any questions on BMJ Open's open peer review process please email info.bmjopen@bmj.com

BMJ Open

Association between social isolation and depressive symptom onset among older adults: A cross national longitudinal study in England and Japan

Journal:	*BMJ Open*
Manuscript ID	bmjopen-2020-045834
Article Type:	Original research
Date Submitted by the Author:	13-Oct-2020
Complete List of Authors:	Noguchi, Taiji; National Center for Geriatrics and Gerontology, Department of Social Science, Center for Gerontology and Social Science; Nagoya City University Graduate School of Medical Sciences and Medical School, Department of Public Health<br>Saito, Masashige; Nihon Fukushi University, Faculty of Social Welfare<br>Aida, Jun; Tokyo Medical and Dental University Graduate School of Medical and Dental Sciences, Department of Oral Health Promotion; Tohoku University, Division for Regional Community Development, Liaison Center for Innovative Dentistry<br>Cable, Noriko; University College London, Epidemiology and Public Health<br>Tsuji, Taishi; University of Tsukuba, Faculty of Health and Sport Sciences; Chiba University, Department of Social Preventive Medical Sciences, Center for Preventive Medical Sciences<br>Koyama, Shihoko; Osaka International Cancer Institute, Cancer Control Center<br>Ikeda, Takaaki; Yamagata University Faculty of Medicine Graduate School of Medical Science, Department of Health Policy Science; Tohoku University Graduate School of Dentistry School of Dentistry, Department of International and Community Oral Health<br>Osaka, Ken; Tohoku University Graduate School of Dentistry School of Dentistry, Department of International and Community Oral Health<br>Kondo, Katsunori; Chiba University, Department of Social Preventive Medical Sciences, Center for Preventive Medical Sciences; National Center for Geriatrics and Gerontology, Department of Gerontological Evaluation, Center for Gerontology and Social Science
Keywords:	EPIDEMIOLOGY, GERIATRIC MEDICINE, Old age psychiatry < PSYCHIATRY, PUBLIC HEALTH, SOCIAL MEDICINE

SCHOLARONE™
Manuscripts

I, the Submitting Author has the right to grant and does grant on behalf of all authors of the Work (as defined in the below author licence), an exclusive licence and/or a non-exclusive licence for contributions from authors who are: i) UK Crown employees; ii) where BMJ has agreed a CC-BY licence shall apply, and/or iii) in accordance with the terms applicable for US Federal Government officers or employees acting as part of their official duties; on a worldwide, perpetual, irrevocable, royalty-free basis to BMJ Publishing Group Ltd ("BMJ") its licensees and where the relevant Journal is co-owned by BMJ to the co-owners of the Journal, to publish the Work in this journal and any other BMJ products and to exploit all rights, as set out in our licence.

The Submitting Author accepts and understands that any supply made under these terms is made by BMJ to the Submitting Author unless you are acting as an employee on behalf of your employer or a postgraduate student of an affiliated institution which is paying any applicable article publishing charge ("APC") for Open Access articles. Where the Submitting Author wishes to make the Work available on an Open Access basis (and intends to pay the relevant APC), the terms of reuse of such Open Access shall be governed by a Creative Commons licence – details of these licences and which Creative Commons licence will apply to this Work are set out in our licence referred to above.

Other than as permitted in any relevant BMJ Author's Self Archiving Policies, I confirm this Work has not been accepted for publication elsewhere, is not being considered for publication elsewhere and does not duplicate material already published. I confirm all authors consent to publication of this Work and authorise the granting of this licence.

For peer review only

Original research

Title

Association between social isolation and depressive symptom onset among older adults: A cross-national longitudinal study in England and Japan

Taiji Noguchi[1,2*], Masashige Saito[3], Jun Aida[4,5], Noriko Cable[6], Taishi Tsuji[7,8], Shihoko Koyama[9], Takaaki Ikeda[10,11], Ken Osaka[11], Katsunori Kondo[1,8]

[1]Center for Gerontology and Social Science, National Center for Geriatrics and Gerontology, Aichi, Japan

[2]Department of Public Health, Nagoya City University Graduate School of Medical Sciences, Aichi, Japan

[3]Faculty of Social Welfare, Nihon Fukushi University, Aichi, Japan

[4]Department of Oral Health Promotion, Graduate School of Medical and Dental Sciences, Tokyo Medical and Dental University, Tokyo, Japan

[5]Division for Regional Community Development, Liaison Center for Innovative Dentistry, Graduate School of Dentistry, Tohoku University, Miyagi, Japan

[6]Department of Epidemiology and Public Health, University College London, London,

For peer review only

UK

[7]Faculty of Health and Sport Sciences, University of Tsukuba, Tokyo, Japan

[8]Department of Social Preventive Medical Sciences, Center for Preventive Medical Sciences, Chiba University, Chiba, Japan

[9]Cancer Control Center, Osaka International Cancer Institute, Osaka, Japan

[10]Department of Health Policy Science, Graduate School of Medical Science, Yamagata University, Yamagata, Japan

[11]Department of International and Community Oral Health, Tohoku University Graduate School of Dentistry, Miyagi, Japan

***Corresponding author**

7-430 Morioka, Obu, Aichi 474-8511, Japan

E-mail: noguchi.taiji0415@gmail.com

Tel: +81-562-46-2311; Fax: +81-562-48-7474

Word count: 2534 words

For peer review only

ABSTRACT

Objective: Social isolation is a risk factor for depressive symptoms in older age. However, little is known about whether its impact varies depending on country-specific cultural contexts of social relationships, such as friendships or family-based relationships. This study examined the association of social isolation with depressive symptom onset in older adults in England, which values friendship-based relationships, and Japan, where family-based relationships are emphasised.

Design: Prospective longitudinal study.

Setting: We utilized data from two ongoing studies: the English Longitudinal Study of Ageing (ELSA) and the Japan Gerontological Evaluation Study (JAGES).

Participants: Older adults aged ≥ 65 years without depressive symptoms at baseline were followed up regarding depressive symptom onset for two years (2010/11 to 2012/13) for the ELSA and 2.5 years (2010/11 to 2013) for the JAGES.

Primary outcome measure: Depressive symptoms were assessed with the Center for Epidemiologic Studies Depression Scale for the ELSA and Geriatric Depression Scale for the JAGES. Multivariable logistic regression analysis was performed to evaluate social isolation using multiple parameters (marital status; interaction with children, relatives, and friends; and social participation).

For peer review only

Results: The data of 3,331 respondents from the ELSA and 33,127 from the JAGES were analysed. Multivariable logistic regression analysis demonstrated that social isolation was significantly associated with depressive symptom onset in both countries. In the ELSA, poor interaction with children marginally affected depressive symptom onset, while in the JAGES, poor interaction with children and no social participation significantly affected depressive symptom onset.

Conclusions: Despite variations in cultural background, social isolation was associated with depressive symptom onset in both England and Japan. Addressing social isolation to safeguard older adults' mental health must be globally prioritised.

For peer review only

Strengths and limitations of this study

- This is the first cross-national longitudinal study to examine the association between social isolation and depressive symptom onset in England, which values friendship-based relationships, and Japan, where family-based relationships are emphasised.
- This study included a large sample of over 3,300 people from England and 33,000 people from Japan aged 65 years and older.
- A limitation of this study is that we cannot make direct comparisons because of variations in cohort follow-up periods and depressive symptom measurement.

71 • Another limitation is the substitution of social support for the evaluation of social contact so as to use the same social isolation assessment scale in both countries.

For peer review only

INTRODUCTION

With population ageing, there is growing worldwide interest in social issues concerning older adults, including social isolation and the deterioration of physical and mental health. Defined as an objective state where an individual has few close relationships or limited contact within a community,[1] social isolation is recognised as a social determinant of health with relevance to mortality,[2] cardiovascular diseases,[3] dementia,[4] and mental health.[5, 6] Social isolation is a major risk factor for mental health problems in older age. Several systematic reviews have shown that social isolation is associated with depressive symptoms,[5, 6] which, in turn, are correlated with unhealthy behaviours and reduced access to material resources.[7] Depressive symptoms, common in later life, are related to adverse health outcomes such as poor quality of life[8] and functional disability.[9] With the high current global burden of depression expected to increase further by 2030,[10] addressing social isolation is an important gerontological issue for protecting mental health in older adults.

The impacts of social isolation on health may vary by country; these differences arise from the culture relating to social networks within and outside the family. A previous cross-national study showed that in the UK, friendship-based social relationships had a significant impact on longevity, whereas in Japan, this impact was associated with family-

For peer review only

based social relationships.[11] Another study comparing American and Japanese older adults suggested that the presence of children was associated with depressive symptoms only among the latter.[12] As Japan has retained the traditionally strong influence of family and kinship systems on society[13] and the expected reciprocity between adult children and their parents remains a dominant feature,[14] the emphasis on family-based relationships is stronger than it is in the West.

Moreover, the social structure surrounding social isolation varies from country to country. Recognising the impact of social isolation on health and economic loss, the UK established the position of 'Minister of Loneliness' in 2018 and is taking a progressive approach to eliminating social isolation.[15] In contrast, Japan, now a super-aged society, is experiencing a rapid increase in the trend of abstaining from marriage and weakening community and neighbourhood relations,[16] leading to a rise in the number of socially isolated people.[17] In Japan, the proportion of people who rarely or never spent time with those close to them was the highest among Organisation for Economic Co-operation and Development countries. In particular, this figure was much higher than in the UK, which has made advances in tackling social isolation (Japan = 15.3%, UK = 5.0%).[18] Owing to differences in the cultural contexts of social relationships and structures, the impact of social isolation on depressive symptoms is expected to vary across countries.

For peer review only

As the association between social isolation and depressive symptoms is often described as bidirectional,[19] longitudinal studies are needed to determine causality. However, previous cross-national comparative studies have only employed cross-sectional designs.[12] Therefore, using longitudinal data from both countries, the present study aims to investigate the association of social isolation with depressive symptom onset in England, where the emphasis is on friendship-based relationships, and Japan, which prioritises familial relationships.

METHODS

Sample

This longitudinal study was conducted using data from two ongoing prospective cohort studies: the English Longitudinal Study of Ageing (ELSA) and the Japan Gerontological Evaluation Study (JAGES). The ELSA targets independent-living older adults aged ≥ 50, while JAGES participants are community-dwelling individuals aged ≥ 65 who are ineligible for long-term healthcare insurance benefits.[20] Details of the ELSA and JAGES can be found elsewhere.[21, 22] For the present analysis, we used the two waves of data that most closely corresponded with the timing of our study: wave 5 (2010/2011) to wave 6 (2012/2013) for the ELSA, and wave 1 (2010/2011) to wave 2 (2013) for the

For peer review only

JAGES. We harmonised the data by including older adults aged ≥ 65, independent in activities of daily living, and without self-reported dementia. Respondents with depressive symptoms at baseline were excluded, and we followed up the onset of depressive symptoms for two years for the ELSA and 2.5 years for the JAGES.

Depressive symptoms

Based on a previous cross-national study,[23] depressive symptoms were measured both at baseline and follow-up using eight items from the Center for Epidemiologic Studies Depression Scale (CES-D) in the ELSA[24] and the Geriatric Depression Scale (GDS-15) in the JAGES.[25] To identify possible depressive cases, the CES-D cut-off was ≥ 4 while that for the GDS-15 was ≥ 5.[24]

Social isolation

Social isolation levels were assessed using the modified Social Isolation Index (SII).[26] The index was computed with respondents given a point if they: (1) were unmarried or living alone, (2) had poor interaction with children (no children or no social support from children), (3) had poor interaction with relatives (no relatives or no social support from relatives), (4) had poor interaction with friends (less than monthly contact or no social

For peer review only

support from friends), and (5) had no social participation (no participation in any social or religious groups). The total possible score ranged from 0 to 5, with higher scores indicating greater social isolation. The participants were categorised into the following five groups based on their scores: 0, 1, 2, 3, and 4–5 points. We used the total score and the scores of the five sub-components as predictive variables.

Covariates

The covariates included age, gender, educational attainment, household equivalised income, present illness, self-rated health, smoking, and drinking. Age was categorised as 65–69, 70–74, 75–79, 80–84, and ≥ 85. Based on the ages of respondents who had completed formal education, the age of final educational attainment was categorised as ≤ 15 years, 16–18 years, and ≥ 19 years. Household equivalised income was classified into quintiles. Present illness was classified as 'yes' or 'no' for cancer, heart disease, and stroke. Self-rated health was dichotomised as 'poor' and 'good'. Smoking and drinking were dichotomised as 'never/past' and 'current'.

Statistical analysis

We analysed the ELSA and JAGES data separately because of differences in research

For peer review only

design, especially sampling approaches. A longitudinal weight was applied to account for survey non-response for the ELSA but not the JAGES as its design does not allow it. First, we calculated descriptive statistics. Second, we conducted a multivariate logistic regression analysis to examine the association between SII score and depressive symptom onset and obtained odds ratios (ORs) and 95% confidence intervals (CIs) for depressive symptom onset. Model 1 was not adjusted for covariates while Model 2 was adjusted for all covariates. Additionally, we analysed the association between SII sub-components and depressive symptom onset, adjusted for all covariates.

To mitigate potential biases resulting from missing information, we used the multiple imputation approach under the missing at random assumption. We generated 20 imputed datasets for the final analysis, which excluded those who met the exclusion criteria and did not respond to the follow-up surveys, using the multiple imputation by chained equations procedure and pooled the results using Rubin's rule.[27]

The significance level was set at $p < 0.05$. We used R (Version 3.5.2 for Windows) for all statistical analyses.

Ethics issues

The ELSA investigators received ethical approval for all waves of the study from the

For peer review only

National Health Service Research Ethics Committees under the National Research and Ethics Services (MREC/01/2/91). The JAGES protocols were approved by the Ethics Committee on the Research of Human Subjects at Nihon Fukushi University (10-05).

Patient and public involvement

No patients were involved in the development of the research question, study design, or data interpretation in this study.

RESULTS

A total of 3,331 ELSA respondents and 33,127 JAGES respondents were included in the final analysis. Their baseline characteristics are presented in Table 1. The mean age (standard deviation) was 73.6 (6.9) years for the ELSA and 72.4 (5.4) years for the JAGES. Regarding SII scores, the ELSA had the largest number of respondents with 0 and 1 points, while the JAGES had the largest number with 2 and 3 points. In the ELSA, respondents who were older, male, less educated, had a lower income, had heart disease, had poor self-rated health, smoked, did not consume much alcohol, and had higher baseline depressive symptom scores had higher SII scores. A similar trend was observed in the JAGES, but here, those who consumed more alcohol had higher SII scores.

For peer review only

Table 1. Respondents' baseline characteristics

	ELSA [a]					JAGES				
	Social Isolation Index score [b]					Social Isolation Index score [b]				
	0	1	2	3	≥ 4	0	1	2	3	≥ 4
	n = 776	n = 906	n = 525	n = 196	n = 47	n = 1402	n = 5981	n = 9723	n = 8735	n = 2176
Age (years), (%)										
65–69	41.0	36.4	29.9	27.6	28.3	38.6	40.5	38.0	37.0	35.8
70–74	26.1	28.3	28.8	29.3	23.6	32.1	32.5	31.6	31.0	30.4
75–79	20.9	17.5	19.3	15.8	20.4	19.2	18.3	19.5	20.3	21.9
80–84	9.1	12.2	12.5	14.5	15.5	8.3	6.7	8.3	9.0	8.8
≥ 85	2.8	5.7	9.5	12.8	12.2	1.9	2.0	2.6	2.7	3.1
Gender, (%)										
Men	50.0	46.1	45.3	51.9	65.8	27.2	35.1	47.0	64.4	66.1
Women	50.0	53.9	54.7	48.1	34.2	72.8	64.9	53.0	35.6	33.9
Educational attainment (years), (%)										
≤ 15	44.1	51.3	52.3	60.9	69.6	42.9	38.1	37.9	38.7	48.6
16–18	35.0	33.8	34.6	27.5	14.1	40.9	40.8	39.3	36.8	31.1
≥ 19	17.8	12.5	11.0	10.3	14.0	15.7	20.4	21.8	23.3	18.5
Missing	3.2	2.4	2.1	1.3	2.3	0.5	0.7	0.9	1.2	1.8
Household equivalised income, (%)										
1st quintile (lowest)	11.4	18.3	23.2	31.2	14.4	7.4	10.9	13.9	15.6	21.3
2nd quintile	21.5	23.7	26.1	24.1	29.6	13.2	13.5	15.0	15.5	16.4
3rd quintile	20.9	21.8	18.9	19.5	27.1	22.5	30.6	30.3	30.6	28.0
4th quintile	21.9	19.9	18.1	16.5	16.6	14.3	13.6	12.7	11.3	9.5
5th quintile (highest)	22.7	15.0	12.7	8.3	12.4	31.5	22.5	19.2	16.8	12.9
Missing	1.5	1.3	0.9	0.4	0.0	11.1	8.9	8.9	10.2	11.9
Cancer, (%)										
No	96.5	95.8	97.1	97.5	96.5	91.0	91.2	90.8	90.1	89.7
Yes	3.4	4.2	2.9	2.5	3.5	3.2	3.1	3.1	3.4	3.7
Missing	0.1	0.0	0.0	0.0	0.0	5.8	5.7	6.1	6.5	6.6
Heart disease, (%)										
No	90.7	86.9	88.4	88.3	77.3	91.0	91.2	90.8	90.1	89.7
Yes	9.3	13.0	11.6	11.7	22.7	3.2	3.1	3.1	3.4	3.7
Missing	0.0	0.1	0.0	0.0	0.0	5.8	5.7	6.1	6.5	6.6
Stroke, (%)										

For peer review only

No	96.3	96.1	97	94.4	96.6	93.3	93.6	93.1	92.5	92.2
Yes	3.7	3.8	3.0	5.6	3.4	0.9	0.8	0.8	1.0	1.2
Missing	0	0.1	0.0	0.0	0.0	5.8	5.7	6.1	6.5	6.6
Self-rated health, (%)										
Good	86.4	79.9	78.8	75.4	71.3	92.0	91.7	90.6	90.2	87.2
Poor	13.6	20.1	21.2	24.6	28.7	7.2	7.7	8.6	9.0	12.2
Missing	0.0	0.0	0.0	0.0	0.0	0.8	0.5	0.8	0.8	0.6
Smoking, (%)										
Never/past	95.5	93.1	89.8	82.9	89.4	86.4	86.1	82.9	81.5	78.4
Current	4.5	6.9	10.2	17.1	10.6	6.4	7.4	9.5	11.3	14.2
Missing	0.0	0.0	0.0	0.0	0.0	7.1	6.5	7.5	7.2	7.3
Drinking, (%)										
Never/past	7.5	11.8	14.9	25.8	17.3	65.0	60.0	56.5	50.4	55.0
Current	91.3	87.0	82.9	72.6	73.6	30.2	35.7	38.6	44.7	40.4
Missing	1.3	1.2	2.2	1.6	9.2	4.8	4.3	4.9	4.9	4.6
CES-D score at baseline, (%)										
0	57.2	52.0	45.5	42.2	54.0					
1	27.3	26.2	28.6	31.8	25.5					
2	9.8	13.8	17.1	11.9	15.1					
3	5.7	8.0	8.8	14.2	5.4					
GDS score at baseline, (%)										
0						20.5	30.4	29.0	26.4	24.3
1						25.5	28.4	28.6	26.9	27.0
2						22.5	20.0	19.8	21.5	21.1
3						17.5	13.1	13.5	14.7	15.9
4						14.1	8.1	9.1	10.5	11.7

201 ELSA, English Longitudinal Study of Ageing; JAGES, Japan Gerontological Evaluation
202 Study; CES-D, Center for Epidemiologic Studies Depression Scale; GDS, Geriatric
203 Depression Scale
204 [a] ELSA data after sampling weight
205 [b] Missing data: ELSA, n = 468; JAGES, n = 5,110

For peer review only

Table 2 presents the description of social isolation and depressive symptom onset. At follow-up, 190 (6.5%) ELSA respondents and 4,456 (13.5%) JAGES respondents exhibited depressive symptom onset. In both studies, higher SII scores were associated with an increased risk of depressive symptom onset. Regarding SII sub-components, ELSA respondents who were unmarried or living alone were more likely to have depressive symptoms, while this was the case with JAGES respondents with no social participation.

For peer review only

213 **Table 2. Description of social isolation status and depressive symptom onset**

		ELSA [a]		JAGES	
		CES-D score at follow-up		GDS score at follow-up	
		< 4	≥ 4	< 5	≥ 5
		n = 2728	n = 190	n = 28671	n = 4456
Social Isolation Index score, (%)					
0		27.5	13.5	4.4	3.0
1		31.2	29.3	18.6	14.3
2		17.9	18.9	29.8	26.3
3		6.5	10.1	26.2	27.5
≥ 4		1.5	2.9	6.1	9.3
Missing		15.4	25.3	14.8	19.7
Social Isolation Index sub-components, (%)					
Unmarried or living alone	No	71.9	58.5	88.3	85.2
	Yes	28.1	41.5	10.2	12.7
	Missing	0.0	0.0	1.4	2.1
Poor interaction with children	No	81.8	73.4	26.6	25.0
	Yes	12.5	14.7	71.7	72.9
	Missing	5.7	11.8	1.7	2.1
Poor interaction with relatives	No	76.3	68.1	41.1	38.7
	Yes	17.3	18.6	54.4	56.0
	Missing	6.4	13.4	4.5	5.4
Poor interaction with friends	No	72.2	66.0	37.1	31.2
	Yes	18.4	21.6	58.2	63.8
	Missing	9.4	12.4	4.6	5.0
No social participation	No	61.7	46.3	75.0	63.9
	Yes	28.4	32.5	13.0	20.0
	Missing	10.0	21.2	11.7	16.1

214 ELSA, English Longitudinal Study of Ageing; JAGES, Japan Gerontological Evaluation
215 Study; CES-D, Center for Epidemiologic Studies Depression Scale; GDS, Geriatric
216 Depression Scale

217 [a] ELSA data after sampling weight

For peer review only

Table 3 depicts the association between SII scores and depressive symptom onset. Multivariable analysis showed that higher SII scores were associated with a higher risk of depressive symptom onset in both studies after adjusting for all covariates. In the ELSA, the OR of depressive symptom onset was significantly higher from a score ≥ one point (OR [95% CI] compared with zero points, one: 1.68 [1.02–2.75], two: 1.77 [1.03–3.05], three: 2.64 [1.37–5.12], ≥ four: 4.01 [1.43–11.22], *p* for trend = 0.015). In the JAGES, as SII scores increased, the OR of depressive symptom onset gradually increased, reaching significance at ≥ three points (OR [95% CI] compared with zero points, one: 1.10 [0.89–1.35], two: 1.15 [0.94–1.40], three: 1.28 [1.04–1.56], ≥ four: 1.48 [1.18–1.85], *p* for trend < 0.001). These results showed almost the same tendency as the complete case analysis without multiple imputation (Supplementary Table 1).

For peer review only

Table 3. Association between social isolation and depressive symptom onset: multivariable logistic regression analysis

	ELSA		JAGES	
	Crude OR (95% CI)	Adjusted OR (95% CI) [a]	Crude OR (95% CI)	Adjusted OR (95% CI) [a]
Social Isolation Index score				
0	1.00 (reference)	1.00 (reference)	1.00 (reference)	1.00 (reference)
1	1.92^{**} (1.19–3.10)	1.68^{*} (1.02–2.75)	1.14 (0.94–1.39)	1.10 (0.89–1.35)
2	2.15^{**} (1.28–3.62)	1.77^{*} (1.03–3.05)	1.32^{**} (1.09–1.60)	1.15 (0.94–1.40)
3	3.19^{***} (1.73–5.90)	2.64^{**} (1.37–5.12)	1.57^{***} (1.30–1.90)	1.28^{*} (1.04–1.56)
≥ 4	3.85^{**} (1.46–10.18)	4.01^{**} (1.43–11.22)	2.26^{***} (1.83–2.79)	1.48^{***} (1.18–1.85)
	p for trend < 0.001	p for trend = 0.015	p for trend < 0.001	p for trend < 0.001

$^{*}\ p < 0.05$; $^{**}\ p < 0.01$; $^{***}\ p < 0.001$

ELSA, English Longitudinal Study of Ageing; JAGES, Japan Gerontological Evaluation Study; CI, confidence interval; OR, odds ratio

[a] Adjusted for age, gender, educational attainment, household equivalised income, present illness (cancer, heart disease, and stroke), self-rated health, smoking, drinking, and depression score at baseline (Center for Epidemiologic Studies Depression Scale for the ELSA and Geriatric Depression Scale for the JAGES).

For peer review only

Table 4 presents the impacts of SII sub-components on depressive symptom onset. In the ELSA, sub-components were not significant, although poor interaction with children was marginally significant (OR [95% CI], with 'none' as the reference; unmarried or living alone: 1.13 [0.80–1.60], poor interaction with children: 1.55 [1.00–2.41], poor interaction with relatives: 1.24 [0.79–1.94], poor interaction with friends: 1.15 [0.77–1.71], no social participation: 1.22 [0.80–1.87]). In the JAGES, poor interaction with children and no social participation were significantly associated with depressive symptom onset after adjusting for all covariates (OR [95% CI], with 'none' as the reference; unmarried or living alone: 1.11 [1.00–1.24], poor interaction with children: 1.09 [1.01–1.19], poor interaction with relatives: 1.04 [0.96–1.12], poor interaction with friends: 1.03 [0.95–1.11], no social participation: 1.28 [1.17–1.40]). These results were similar to those obtained from the complete case analysis (Supplementary Table 2).

For peer review only

247 **Table 4. Association between sub-components of social isolation and depressive symptom onset: multivariable logistic regression**
248 **analysis**

	ELSA	JAGES
	Adjusted OR (95% CI) [a]	Adjusted OR (95% CI) [a]
Social Isolation Index sub-components (reference: none)		
Unmarried or living alone	1.13 (0.80–1.60)	1.11† (1.00–1.24)
Poor interaction with children	1.55† (1.00–2.41)	1.09* (1.01–1.19)
Poor interaction with relatives	1.24 (0.79–1.94)	1.04 (0.96–1.12)
Poor interaction with friends	1.15 (0.77–1.71)	1.03 (0.95–1.11)
No social participation	1.22 (0.80–1.87)	1.28*** (1.17–1.40)

249 * $p < 0.05$; ** $p < 0.01$; *** $p < 0.001$; † $p < 0.1$

250 ELSA, English Longitudinal Study of Ageing; JAGES, Japan Gerontological Evaluation Study; CI, confidence interval; OR, odds ratio

251 [a] Adjusted for age, gender, educational attainment, equivalent income, present illness (cancer, heart disease, and stroke), self-rated health,
252 smoking, drinking, and depression score at baseline (Center for Epidemiologic Studies Depression Scale for the ELSA and Geriatric
253 Depression Scale for the JAGES).

For peer review only

DISCUSSION

To the best of our knowledge, this is the first cross-national longitudinal study of the association of social isolation with depressive symptoms among older English and Japanese adults. Social isolation was significantly associated with depressive symptom onset in both countries. Our results support previous longitudinal findings on social relationships and mental health in older adults in England[28] and Japan.[29] Using data frames that were similar with regard to assessment and covariates, we showed that social isolation is a common risk factor for depressive symptoms in England and Japan despite cultural differences such as emphasis on friendships versus familism, respectively. Thus, our results suggest that to safeguard the mental health of older adults, addressing social isolation is a global need.

The association between social isolation and depressive symptoms was somewhat stronger in England than in Japan. Although we cannot make direct comparisons because of variations in cohort follow-up periods and depressive symptom measurement, there are several possible reasons for this pattern of findings. The impact of social factors could be different depending on the group and society to which one belongs. This is best understood in the context of the concept of relative deprivation.[30] In other words, higher levels of relative social isolation may induce greater psychological

For peer review only

stress. A previous study showed that rich community ties and cohesion were protective factors for health but could have a negative effect on those who were not socially involved.[31] Being isolated in a connected society such as England may represent a more severe condition, which may have a stronger negative impact on mental health.

While a previous study showed that friendship-based relationships contributed to longevity in older adults in England, in the present study they were not associated with depressive symptom onset. Another study focused on English older adults indicated the contribution of partner and child support, rather than support from friends, in alleviating depression;[32] thus, relationships with family members might be important for mental health. In the JAGES, poor interaction with children and no social participation were significantly associated with depressive symptom onset. Previous studies on older Japanese adults suggested that interaction with children and social participation were protective factors for mental health problems.[12, 33] Traditionally, Japan has had strong family and kinship ties, and adult children are expected to demonstrate reciprocity with their parents.[14] However, children now tend to live apart from their parents after marriage, and contact is reduced,[34] potentially leading to loneliness in older adults. In such a situation, social participation may improve mental health by establishing a sense of belonging.[33] Our results demonstrate that promoting interaction with children and

For peer review only

community participation are essential for safeguarding mental health in older Japanese adults.

This study has several strengths. First, it is the first cross-national population-level investigation of the impact of social isolation on depressive symptom onset using a unified data frame. Second, by using two longitudinal datasets, we were able to determine causality in the association between social isolation and depressive symptoms. Third, the use of large-scale data allowed us to detect the effects of relatively rare situations of severe social isolation.

However, certain limitations cannot be ignored. First, the measurement of depressive symptoms in the two cohorts was not the same. Therefore, we could not directly compare depressive symptom onset in the two countries. However, these measurements were also used in a previous cross-national comparison study in England and Japan,[23] and we were able to examine the association between social isolation and depressive symptom onset in each country using the same data frame. Second, we substituted social support for the assessment of social contact for some items in order to use the same SII. Therefore, cultural differences in expectations regarding the receipt of social support in both countries might have caused information biases. For instance, expectations regarding social support from relatives could originally have been higher in

For peer review only

Japan,[14] leading to overestimation of social isolation levels. Third, there were differences in study design in the data from the two cohorts, such as sampling method and follow-up period. However, we made efforts to harmonise the data: those aged ≤ 64, with dementia, and dependent in activities of daily living were excluded from the analysis. Also, the ELSA presents nationally representative population data, while the JAGES does not. However, the JAGES sample is representative of areas from a nationwide ageing study in which about one-fifth of all prefectures (nine out of 47) were enrolled.

CONCLUSION

We examined the association between social isolation and depressive symptom onset among older adults in England and Japan, which have different cultural contexts regarding social relationships, finding a significant association in both countries. Thus, globally, tackling social isolation must be prioritised to safeguard the mental health of older adults. Particularly in Japan, the promotion of interaction with children and social participation could be key factors in addressing social isolation.

ACKNOWLEDGEMENTS

We wish to express our deepest gratitude to the members in the Japan Gerontological

For peer review only

Evaluation Study. We would also like to thank everyone who participated in the surveys.

AUTHORS' CONTRIBUTIONS

All authors contributed to the conception and design of this study. Data collection was primarily conducted by MS, JA, NC, KO, and KK. Analyses were performed by TN, MS, JA, TT, SK, and TI. TN prepared the initial manuscript and MS, JA, NC, TT, SK, TI, KO, and KK significantly contributed to revising it. All authors read and approved the final manuscript.

FUNDING STATEMENT

This work was supported by a grant from the Grants-in-Aid for Scientific Research (18KK0057, 19K24277) from the Japan Society for the Promotion of Science. The English Longitudinal Study of Ageing was developed by a team of researchers based at the University College London, NatCen Social Research, and the Institute for Fiscal Studies. The data were collected by NatCen Social Research. The funding is currently provided by the National Institute of Aging (R01AG017644) and a consortium of UK government departments coordinated by the National Institute for Health Research. The Japan Gerontological Evaluation Study is supported by the Ministry of Education, Culture,

For peer review only

Sports, Science and Technology-Japan-Supported Program for the Strategic Research Foundation at Private Universities (2009-2013), Japan Society for the Promotion of Science KAKENHI Grant Numbers (JP18390200, JP22330172, JP22390400, JP23243070, JP23590786, JP23790710, JP24390469, JP24530698, JP24683018, JP25253052, JP25870573, JP25870881, JP26285138, JP26882010, JP15H01972), Health Labour Sciences Research Grants (H22-Choju-Shitei-008, H24-Junkanki -Ippan-007, H24-Chikyukibo-Ippan-009, H24-Choju-Wakate-009, H25-Kenki-Wakate-015, H25-Choju-Ippan-003, H26-Irryo-Shitei-003 [Fukkou], H26-Choju-Ippan-006, H27-Ninchisyou-Ippan-001, H28-chojuIppan-002, H28-Ninchisho-Ippan-002, H30-Kenki-Ippan-006, H30-Junkankitou-Ippan-004), Japan Agency for Medical Research and Development (JP17dk0110017, JP18dk0110027, JP18ls0110002, JP18le0110009, JP19dk0110034), Research Funding for Longevity Sciences from the National Center for Geriatrics and Gerontology (24-17, 24-23, 29-42, 30-22, 20-40), Open Innovation Platform with Enterprises, and Research Institute and Academia (OPERA, JPMJOP1831) from the Japan Science and Technology Agency. The funding sources had no role in the study design, data collection and analysis, decision to publish or preparation of the manuscript.

For peer review only

DATA SHARING STATEMENT

For the JAGES, all enquiries are to be addressed to the data management committee via e-mail: dataadmin.ml@jages.net. All JAGES datasets have ethical or legal restrictions for public deposition because of the inclusion of sensitive information about the human participants. Regarding the ELSA, data are available in an open-access repository at http://www. dataarchive.ac.uk/.

COMPETING INTERESTS STATEMENT

The authors declare no conflict of interest.

REFERENCES

1. Townsend P. The family life of old people: an investigation in East London. *Sociol Rev* 1955;3:175–95. doi: 10.1111/j.1467-954X.1955.tb01052.x

2. Holt-Lunstad J, Smith TB, Layton JB. Social relationships and mortality risk: a meta-analytic review. *PLoS Med* 2010;7:e1000316. doi:10.1371/journal.pmed.1000316

3. Valtorta NK, Kanaan M, Gilbody S, et al. Loneliness and social isolation as risk factors for coronary heart disease and stroke: systematic review and meta-analysis of longitudinal observational studies. *Heart* 2016;102:1009–16.

For peer review only

doi:10.1136/heartjnl-2015-308790

4. Livingston G, Sommerlad A, Orgeta V, et al. Dementia prevention, intervention, and care. *Lancet* 2017;390:2673–734. doi:10.1016/s0140-6736(17)31363-6

5. Leigh-Hunt N, Bagguley D, Bash K, et al. An overview of systematic reviews on the public health consequences of social isolation and loneliness. *Public Health* 2017;152:157–71. doi:10.1016/j.puhe.2017.07.035

6. Santini ZI, Koyanagi A, Tyrovolas S, et al. The association between social relationships and depression: a systematic review. *J Affect Disord* 2015;175:53–65. doi:10.1016/j.jad.2014.12.049

7. Kawachi I, Berkman LF. Social ties and mental health. *J Urban Health* 2001;78:458–67. doi:10.1093/jurban/78.3.458

8. Sivertsen H, Bjorklof GH, Engedal K, et al. Depression and quality of life in older persons: a review. *Dement Geriatr Cogn Disord* 2015;40:311–39. doi:10.1159/000437299

9. Chen CM, Mullan J, Su YY, et al. The longitudinal relationship between depressive symptoms and disability for older adults: a population-based study. *J Gerontol A Biol Sci Med Sci* 2012;67:1059–67. doi:10.1093/gerona/gls074

10. World Federation for Mental Health. Depression: a global crisis. World Mental Health

For peer review only

Day, October 10 2012. 2012. https://www.who.int/mental_health/management/depression/wfmh_paper_depression_wmhd_2012.pdf (accessed 7 Feb 2020).

11. Aida J, Cable N, Zaninotto P, et al. Social and behavioural determinants of the difference in survival among older adults in Japan and England. *Gerontology* 2018;64:266–77. doi:10.1159/000485797

12. Sugisawa H, Shibata H, Hougham GW, et al. The impact of social ties on depressive symptoms in U.S. and Japanese elderly. *J Soc Issues* 2003;58:785–804. doi:10.1111/1540-4560.00290

13. Takagi E, Silverstein M. Intergenerational coresidence of the Japanese elderly: are cultural norms proactive or reactive? 2006;28:473–92. doi:10.1177/0164027506287788

14. Brown JW, Liang J, Krause N, et al. Transitions in living arrangements among elders in Japan: does health make a difference? *J Gerontol B Psychol Sci Soc Sci* 2002;57:S209–20. doi:10.1093/geronb/57.4.s209

15. HM Government. A connected society: a strategy for tackling loneliness – laying the foundations for change. 2018. https://assets.publishing.service.gov.uk/government/uploads/system/uploads/atta

For peer review only

chment_data/file/750909/6.4882_DCMS_Loneliness_Strategy_web_Update.pdf (accessed 12 October 2020).

16. National Institute of Population and Social Security Research. Household projections for Japan. http://www.ipss.go.jp/pp-ajsetai/e/hprj_98/hprj_98.html (accessed 12 October 2020).

17. Murayama H, Shibui Y, Fukuda Y, et al. A new crisis in Japan-social isolation in old age. *J Am Geriatr Soc* 2011;59:2160–2. doi:10.1111/j.1532-5415.2011.03640.x

18. Organisation for Economic Co-operation and Development. Society at a Glance: OECD Social Indicators. Paris: OECD Publishing 2005.

19. Santini ZI, Jose PE, York Cornwell E, et al. Social disconnectedness, perceived isolation, and symptoms of depression and anxiety among older Americans (NSHAP): a longitudinal mediation analysis. *Lancet Public Health* 2020;5:e62–70. doi:10.1016/s2468-2667(19)30230-0

20. Mossialos E, Djordjevic A, Osborn R, et al. International profiles of health care systems. 2017. https://www.commonwealthfund.org/sites/default/files/documents/___media_files_publications_fund_report_2017_may_mossialos_intl_profiles_v5.pdf (accessed 12 October 2020).

For peer review only

21. Kondo K. Progress in aging epidemiology in Japan: the JAGES Project. *J Epidemiol* 2016;26:331–6. doi:10.2188/jea.JE20160093

22. Zhao Y, Hu Y, Smith JP, et al. Cohort profile: the China Health and Retirement Longitudinal Study (CHARLS). *Int J Epidemiol* 2014;43:61–8. doi:10.1093/ije/dys203

23. Cable N, Chandola T, Aida J, et al. Can sleep disturbance influence changes in mental health status? Longitudinal research evidence from ageing studies in England and Japan. *Sleep Med* 2017;30:216–21. doi:10.1016/j.sleep.2016.11.017

24. Gallagher D, Kiss A, Lanctot K, et al. Depressive symptoms and cognitive decline: a longitudinal analysis of potentially modifiable risk factors in community dwelling older adults. *J Affect Disord* 2016;190:235–40. doi:10.1016/j.jad.2015.09.046

25. Burke WJ, Roccaforte WH, Wengel SP. The short form of the Geriatric Depression Scale: a comparison with the 30-item form. *J Geriatr Psychiatry Neurol* 1991;4:173–8. doi:10.1177/089198879100400310

26. Shankar A, McMunn A, Banks J, et al. Loneliness, social isolation, and behavioral and biological health indicators in older adults. *Health Psychol* 2011;30:377–85. doi:10.1037/a0022826

27. White IR, Royston P, Wood AM. Multiple imputation using chained equations: issues

For peer review only

452 and guidance for practice. *Stat Med* 2011;30:377–99. doi:10.1002/sim.4067

453 28. Cruwys T, Dingle GA, Haslam C, et al. Social group memberships protect against
454 future depression, alleviate depression symptoms and prevent depression relapse.
455 *Soc Sci Med* 2013;98:179–86. doi:10.1016/j.socscimed.2013.09.013

456 29. Koizumi Y, Awata S, Kuriyama S, et al. Association between social support and
457 depression status in the elderly: results of a 1-year community-based prospective
458 cohort study in Japan. *Psychiatry Clin Neurosci* 2005;59:563–9.
459 doi:10.1111/j.1440-1819.2005.01415.x

460 30. Smith HJ, Pettigrew TF, Pippin GM, et al. Relative deprivation: a theoretical and
461 meta-analytic review. *Pers Soc Psychol Rev* 2012;16:203–32.
462 doi:10.1177/1088868311430825

463 31. Amemiya A, Saito J, Saito M, et al. Social capital and the improvement in functional
464 ability among older people in Japan: a multilevel survival analysis using JAGES
465 data. *Int J Environ Res Public Health* 2019;16:1310. doi:10.3390/ijerph16081310

466 32. Stafford M, McMunn A, Zaninotto P, et al. Positive and negative exchanges in social
467 relationships as predictors of depression: evidence from the English Longitudinal
468 Study of Aging. *J Aging Health* 2011;23:607–28.
469 doi:10.1177/0898264310392992

For peer review only

470 33. Takagi D, Kondo K, Kawachi I. Social participation and mental health: moderating effects of gender, social role and rurality. *BMC Public Health* 2013;13:701. doi:10.1186/1471-2458-13-701

473 34. Takagi E, Silverstein M. Purchasing piety? Coresidence of married children with their older parents in Japan. *Demography* 2011;48:1559–79. doi:10.1007/s13524-011-0053-0

For peer review only

Supplementary Table 1. Association between social isolation and depressive symptom onset, by complete case analysis

		ELSA		JAGES	
		Crude OR (95% CI)	Adjusted OR (95% CI)	Crude OR (95% CI)	Adjusted OR (95% CI)
Social Isolation Index score	0	1.00 (reference)	1.00 (reference)	1.00 (reference)	1.00 (reference)
	1	1.92** (1.19–3.10)	1.56† (0.94–2.60)	1.14 (0.94–1.39)	1.11 (0.87–1.41)
	2	2.15** (1.28–3.62)	1.71† (0.98–2.99)	1.32** (1.09–1.60)	1.20 (0.95–1.51)
	3	3.19*** (1.73–5.90)	2.50** (1.27–4.90)	1.57*** (1.30–1.90)	1.30* (1.03–1.64)
	≥ 4	3.85** (1.46–10.18)	5.17** (1.83–14.66)	2.26*** (1.83–2.79)	1.47** (1.13–1.91)
		p for trend < 0.001	p for trend < 0.001	p for trend < 0.001	p for trend < 0.001
Age (years)	65–69		1.00 (reference)		1.00 (reference)
	70–74		1.37 (0.83–2.25)		1.16** (1.05–1.30)
	75–79		1.24 (0.71–2.14)		1.31*** (1.16–1.48)
	80–84		1.66† (0.92–2.99)		1.60*** (1.37–1.88)
	≥ 85		0.97 (0.45–2.09)		1.79*** (1.39–2.30)
Gender	Male		1.00 (reference)		1.00 (reference)
	Female		1.58* (1.07–2.34)		1.00 (0.90–1.11)
Educational attainment (years)	< 15		1.00 (reference)		1.00 (reference)
	16–18		1.05 (0.68–1.60)		0.81*** (0.74–0.89)
	≥ 19		1.30 (0.70–2.41)		0.68*** (0.60–0.78)
Household equivalised income	1st quintile (lowest)		1.00 (reference)		1.00 (reference)
	2nd quintile		0.70 (0.40–0.21)		0.95 (0.83–1.09)
	3rd quintile		1.11 (0.66–1.87)		0.76*** (0.67–0.87)
	4th quintile		0.82 (0.45–1.50)		0.67*** (0.57–0.79)
	5th quintile (highest)		0.84 (0.42–1.68)		0.65*** (0.56–0.76)

For peer review only

Cancer	No	1.00 (reference)	1.00 (reference)
	Yes	0.95 (0.32–2.81)	1.16 (0.93–1.43)
Heart disease	No	1.00 (reference)	1.00 (reference)
	Yes	1.13 (0.67–1.92)	1.20 (1.05–1.37)
Stroke	No	1.00 (reference)	1.00 (reference)
	Yes	1.3 (0.56–3.02)	1.18 (0.80–1.73)
Self-rated health	Good	1.00 (reference)	1.00 (reference)
	Poor	1.71* (1.13–2.59)	1.63*** (1.44–1.85)
CES-D score at baseline		1.90*** (1.60–2.25)	
GDS score at baseline			1.92*** (1.86–1.99)
Smoking	Never/past	1.00 (reference)	1.00 (reference)
	Current	0.76 (0.76–1.56)	1.29*** (1.12–1.48)
Drinking	Never/past	1.00 (reference)	1.00 (reference)
	Current	1.41 (0.81 – 2.44)	1.02 (0.93 – 1.13)

*, $p < 0.05$; **, $p < 0.01$; ***, $p < 0.001$; †, $p < 0.1$

ELSA, English Longitudinal Study of Ageing; JAGES, Japan Gerontological Evaluation Study; CES-D, Center for Epidemiologic Studies Depression Scale; CI, confidence interval; GDS, Geriatric Depression Scale; OR, odds ratio

For peer review only

Supplementary Table 2. Association between sub-components of social isolation and depressive symptom onset, by complete case analysis

		ELSA		JAGES	
		Crude OR (95% CI)	Adjusted OR (95% CI)	Crude OR (95% CI)	Adjusted OR (95% CI)
Social Isolation Index sub-components (ref; none)					
Unmarried or living alone		1.91*** (1.34–2.72)	1.27 (0.84–1.93)	1.33*** (1.18–1.49)	1.02 (0.88–1.19)
Poor interaction with children		1.08 (0.68–1.74)	1.51 (0.91–2.50)	1.01 (0.98–1.10)	1.13* (1.02–1.25)
Poor interaction with relatives		1.25 (0.83–1.90)	1.33 (0.85–2.08)	1.05 (0.97–1.13)	0.98 (0.90–1.08)
Poor interaction with friends		1.19 (0.78–1.80)	1.21 (0.78–1.87)	1.23 (1.14–1.33)	1.11* (1.01–1.22)
No social participation		1.54* (1.08–2.19)	1.46† (0.98–2.16)	1.70 (1.56–1.86)	1.22*** (1.09–1.36)
Age (years)	65–69		1.00 (reference)		1.00 (reference)
	70–74		1.37 (0.83–2.25)		1.17** (1.05–1.30)
	75–79		1.25 (0.72–2.17)		1.31*** (1.16–1.49)
	80–84		1.68† (0.92–3.04)		1.60*** (1.36–1.88)
	≥ 85		1.00 (0.46–2.21)		1.78*** (1.38–2.30)
Gender	Male		1.00 (reference)		1.00 (reference)
	Female		1.59* (1.07–2.37)		1.00 (0.89–1.11)
Educational attainment (years)	< 15		1.00 (reference)		1.00 (reference)
	10–12		1.04 (0.67–1.60)		0.81*** (0.74–0.90)
	≥ 19		1.31 (0.70–2.46)		0.69*** (0.61–0.78)
Household equivalised income	1st quintile (lowest)		1.00 (reference)		1.00 (reference)
	2nd quintile		0.71 (0.41–1.23)		0.95 (0.83–1.09)
	3rd quintile		1.13 (0.67–1.93)		0.76*** (0.67–0.86)
	4th quintile		0.83 (0.45–1.52)		0.67*** (0.57–0.79)
	5th quintile (highest)		0.85 (0.42–1.71)		0.66*** (0.57–0.76)
Cancer	No		1.00 (reference)		1.00 (reference)
	Yes		0.95 (0.32–2.81)		1.15 (0.93–1.43)

For peer review only

Heart disease	No	1.00 (reference)	1.00 (reference)
	Yes	1.16 (0.69–1.96)	1.20** (1.05–1.37)
Stroke	No	1.00 (reference)	1.00 (reference)
	Yes	1.28 (0.55–2.99)	1.18 (0.80–1.73)
Self-rated health	Good	1.00 (reference)	1.00 (reference)
	Poor	1.71* (1.13–2.58)	1.62*** (1.43–1.83)
CES-D score at baseline		1.89*** (1.59–2.25)	1.00 (reference)
GDS score at baseline			1.92*** (1.85–1.99)
Smoking	Never/past	1.00 (reference)	1.00 (reference)
	Current	0.74 (0.36–1.53)	1.29*** (1.13–1.48)
Drinking	Never/past	1.00 (reference)	1.00 (reference)
	Current	1.39 (0.80–2.40)	1.03 (0.93–1.14)

*, $p < 0.05$; **, $p < 0.01$; ***, $p < 0.001$; †, $p < 0.1$

ELSA, English Longitudinal Study of Ageing; JAGES, Japan Gerontological Evaluation Study; CES-D, Center for Epidemiologic Studies Depression Scale; CI, confidence interval; GDS, Geriatric Depression Scale; OR, odds ratio

For peer review only

STROBE Statement—Checklist of items that should be included in reports of ***cohort studies***

	Item No	Recommendation	Page No
**Title and abstract**	1	(*a*) Indicate the study's design with a commonly used term in the title or the abstract	1, 3
		(*b*) Provide in the abstract an informative and balanced summary of what was done and what was found	3-4
**Introduction**			
Background/rationale	2	Explain the scientific background and rationale for the investigation being reported	6-8
Objectives	3	State specific objectives, including any prespecified hypotheses	8
**Methods**			
Study design	4	Present key elements of study design early in the paper	8
Setting	5	Describe the setting, locations, and relevant dates, including periods of recruitment, exposure, follow-up, and data collection	8-9
Participants	6	(*a*) Give the eligibility criteria, and the sources and methods of selection of participants. Describe methods of follow-up	8-9
		(*b*) For matched studies, give matching criteria and number of exposed and unexposed	Not applicable
Variables	7	Clearly define all outcomes, exposures, predictors, potential confounders, and effect modifiers. Give diagnostic criteria, if applicable	9-10
Data sources/ measurement	8*	For each variable of interest, give sources of data and details of methods of assessment (measurement). Describe comparability of assessment methods if there is more than one group	9-10
Bias	9	Describe any efforts to address potential sources of bias	11
Study size	10	Explain how the study size was arrived at	Not applicable
Quantitative variables	11	Explain how quantitative variables were handled in the analyses. If applicable, describe which groupings were chosen and why	9-10
Statistical methods	12	(*a*) Describe all statistical methods, including those used to control for confounding	10-11
		(*b*) Describe any methods used to examine subgroups and interactions	Not applicable
		(*c*) Explain how missing data were addressed	11
		(*d*) If applicable, explain how loss to follow-up was addressed	Not applicable
		(*e*) Describe any sensitivity analyses	Not applicable
**Results**			
Participants	13*	(a) Report numbers of individuals at each stage of study—eg numbers potentially eligible, examined for eligibility, confirmed eligible, included in the study, completing follow-up, and analysed	12-14
		(b) Give reasons for non-participation at each stage	Not applicable
		(c) Consider use of a flow diagram	Not applicable
Descriptive data	14*	(a) Give characteristics of study participants (eg demographic, clinical, social) and information on exposures and potential confounders	12-14
		(b) Indicate number of participants with missing data for each variable of interest	13-14
		(c) Summarise follow-up time (eg, average and total amount)	9
Outcome data	15*	Report numbers of outcome events or summary measures over time	15-16

For peer review only

Main results	16	(*a*) Give unadjusted estimates and, if applicable, confounder-adjusted estimates and their precision (eg, 95% confidence interval). Make clear which confounders were adjusted for and why they were included	17-18
		(*b*) Report category boundaries when continuous variables were categorized	Not applicable
		(*c*) If relevant, consider translating estimates of relative risk into absolute risk for a meaningful time period	Not applicable
Other analyses	17	Report other analyses done—eg analyses of subgroups and interactions, and sensitivity analyses	Supplementary 1, 2
**Discussion**			
Key results	18	Summarise key results with reference to study objectives	21
Limitations	19	Discuss limitations of the study, taking into account sources of potential bias or imprecision. Discuss both direction and magnitude of any potential bias	23-24
Interpretation	20	Give a cautious overall interpretation of results considering objectives, limitations, multiplicity of analyses, results from similar studies, and other relevant evidence	21-23
Generalisability	21	Discuss the generalisability (external validity) of the study results	Not applicable
**Other information**			
Funding	22	Give the source of funding and the role of the funders for the present study and, if applicable, for the original study on which the present article is based	25-26

*Give information separately for exposed and unexposed groups.

Note: An Explanation and Elaboration article discusses each checklist item and gives methodological background and published examples of transparent reporting. The STROBE checklist is best used in conjunction with this article (freely available on the Web sites of PLoS Medicine at http://www.plosmedicine.org/, Annals of Internal Medicine at http://www.annals.org/, and Epidemiology at http://www.epidem.com/). Information on the STROBE Initiative is available at http://www.strobe-statement.org.

For peer review only

BMJ Open

Association between social isolation and depression onset among older adults: A cross-national longitudinal study in England and Japan

Journal:	*BMJ Open*
Manuscript ID	bmjopen-2020-045834.R1
Article Type:	Original research
Date Submitted by the Author:	11-Feb-2021
Complete List of Authors:	Noguchi, Taiji; National Center for Geriatrics and Gerontology, Department of Social Science, Center for Gerontology and Social Science; Nagoya City University Graduate School of Medical Sciences and Medical School, Department of Public Health<br>Saito, Masashige; Nihon Fukushi University, Faculty of Social Welfare<br>Aida, Jun; Tokyo Medical and Dental University Graduate School of Medical and Dental Sciences, Department of Oral Health Promotion; Tohoku University, Division for Regional Community Development, Liaison Center for Innovative Dentistry<br>Cable, Noriko; University College London, Epidemiology and Public Health<br>Tsuji, Taishi; University of Tsukuba, Faculty of Health and Sport Sciences; Chiba University, Department of Social Preventive Medical Sciences, Center for Preventive Medical Sciences<br>Koyama, Shihoko; Osaka International Cancer Institute, Cancer Control Center<br>Ikeda, Takaaki; Yamagata University Faculty of Medicine Graduate School of Medical Science, Department of Health Policy Science; Tohoku University Graduate School of Dentistry School of Dentistry, Department of International and Community Oral Health<br>Osaka, Ken; Tohoku University Graduate School of Dentistry School of Dentistry, Department of International and Community Oral Health<br>Kondo, Katsunori; Chiba University, Department of Social Preventive Medical Sciences, Center for Preventive Medical Sciences; National Center for Geriatrics and Gerontology, Department of Gerontological Evaluation, Center for Gerontology and Social Science
**Primary Subject Heading**:	Epidemiology
Secondary Subject Heading:	Mental health, Geriatric medicine, Public health
Keywords:	EPIDEMIOLOGY, GERIATRIC MEDICINE, Old age psychiatry < PSYCHIATRY, PUBLIC HEALTH, SOCIAL MEDICINE

SCHOLARONE™
Manuscripts

I, the Submitting Author has the right to grant and does grant on behalf of all authors of the Work (as defined in the below author licence), an exclusive licence and/or a non-exclusive licence for contributions from authors who are: i) UK Crown employees; ii) where BMJ has agreed a CC-BY licence shall apply, and/or iii) in accordance with the terms applicable for US Federal Government officers or employees acting as part of their official duties; on a worldwide, perpetual, irrevocable, royalty-free basis to BMJ Publishing Group Ltd ("BMJ") its licensees and where the relevant Journal is co-owned by BMJ to the co-owners of the Journal, to publish the Work in this journal and any other BMJ products and to exploit all rights, as set out in our licence.

The Submitting Author accepts and understands that any supply made under these terms is made by BMJ to the Submitting Author unless you are acting as an employee on behalf of your employer or a postgraduate student of an affiliated institution which is paying any applicable article publishing charge ("APC") for Open Access articles. Where the Submitting Author wishes to make the Work available on an Open Access basis (and intends to pay the relevant APC), the terms of reuse of such Open Access shall be governed by a Creative Commons licence – details of these licences and which Creative Commons licence will apply to this Work are set out in our licence referred to above.

Other than as permitted in any relevant BMJ Author's Self Archiving Policies, I confirm this Work has not been accepted for publication elsewhere, is not being considered for publication elsewhere and does not duplicate material already published. I confirm all authors consent to publication of this Work and authorise the granting of this licence.

For peer review only

Original research

Title

Association between social isolation and depression onset among older adults: A cross-national longitudinal study in England and Japan

Taiji Noguchi[1,2*], Masashige Saito[3], Jun Aida[4,5], Noriko Cable[6], Taishi Tsuji[7,8], Shihoko Koyama[9], Takaaki Ikeda[10,11], Ken Osaka[11], Katsunori Kondo[1,8]

[1]Center for Gerontology and Social Science, National Center for Geriatrics and Gerontology, Aichi, Japan

[2]Department of Public Health, Nagoya City University Graduate School of Medical Sciences, Aichi, Japan

[3]Faculty of Social Welfare, Nihon Fukushi University, Aichi, Japan

[4]Department of Oral Health Promotion, Graduate School of Medical and Dental Sciences, Tokyo Medical and Dental University, Tokyo, Japan

[5]Division for Regional Community Development, Liaison Center for Innovative Dentistry, Graduate School of Dentistry, Tohoku University, Miyagi, Japan

[6]Department of Epidemiology and Public Health, University College London, London,

For peer review only

UK

[7]Faculty of Health and Sport Sciences, University of Tsukuba, Tokyo, Japan

[8]Department of Social Preventive Medical Sciences, Center for Preventive Medical Sciences, Chiba University, Chiba, Japan

[9]Cancer Control Center, Osaka International Cancer Institute, Osaka, Japan

[10]Department of Health Policy Science, Graduate School of Medical Science, Yamagata University, Yamagata, Japan

[11]Department of International and Community Oral Health, Tohoku University Graduate School of Dentistry, Miyagi, Japan

***Corresponding author**

7-430 Morioka, Obu, Aichi 474-8511, Japan

E-mail: noguchi.taiji0415@gmail.com

Tel: +81-562-46-2311; Fax: +81-562-48-7474

Word count: 3,199 words

For peer review only

ABSTRACT

Objective: Social isolation is a risk factor for depression in older age. However, little is known about whether its impact varies depending on country-specific cultural contexts regarding social relationships. The present study examined the association of social isolation with depression onset among older adults in England, which has taken advanced measures against social isolation, and Japan, a super-aged society with a rapidly increasing number of socially isolated people.

Design: Prospective longitudinal study.

Setting: We utilised data from two ongoing studies: the English Longitudinal Study of Ageing (ELSA) and the Japan Gerontological Evaluation Study (JAGES).

Participants: Older adults aged ≥ 65 years without depression at baseline were followed up regarding depression onset for two years (2010/11 to 2012/13) for the ELSA and 2.5 years (2010/11 to 2013) for the JAGES.

Primary outcome measure: Depression was assessed with the Center for Epidemiologic Studies Depression Scale for the ELSA and Geriatric Depression Scale for the JAGES. Multivariable logistic regression analysis was performed to evaluate social isolation using multiple parameters (marital status; interaction with children, relatives, and friends; and social participation).

For peer review only

Results: The data of 3,331 respondents from the ELSA and 33,127 from the JAGES were analysed. Multivariable logistic regression analysis demonstrated that social isolation was significantly associated with depression onset in both countries. In the ELSA, poor interaction with children was marginally associated with depression onset, while in the JAGES, poor interaction with children and no social participation significantly affected depression onset.

Conclusions: Despite variations in cultural background, social isolation was associated with depression onset in both England and Japan. Addressing social isolation to safeguard older adults' mental health must be globally prioritised.

Strengths and limitations of this study

- This is the first cross-national longitudinal study to examine the association between social isolation and depression onset in England, which has taken advanced measures against social isolation, and Japan, a super-aged society with a rapidly increasing number of socially isolated people.
- This study included a large sample of over 3,300 people from England and 33,000 people from Japan aged 65 years and older.
- A limitation of this study is that we cannot make direct comparisons because of

For peer review only

variations in cohort follow-up periods and depression measurement.

- Another limitation is the use of social support for the evaluation of social contact so as to be able to use the same social isolation assessment scale in both countries.

For peer review only

INTRODUCTION

With population ageing, there is growing worldwide interest in social issues concerning older adults, including social isolation and the deterioration of physical and mental health. Defined as an objective state where an individual has few close relationships or limited contact within a community,[1] social isolation is recognised as a social determinant of health with relevance to mortality,[2] cardiovascular diseases,[3] dementia,[4] and mental health.[5, 6] Social isolation is a major risk factor for mental health problems in older age. Several systematic reviews have shown that social isolation is associated with depressive symptoms,[5, 6] which, in turn, are correlated with unhealthy behaviours and reduced access to material resources.[7] Depression, common in later life, is related to adverse health outcomes such as poor quality of life[8] and functional disability.[9] With the high current global burden of depression expected to increase further by 2030,[10] addressing social isolation is an important gerontological issue for protecting mental health in older adults.

The impacts of social isolation on health may vary by country; this could be the result of differences in the social environments related to social networks within and outside the family. A recent study of older adults in England and Japan showed that social isolation is a common risk factor for mortality in both countries, with a greater impact in

For peer review only

England; the results are discussed in terms of possible differences between societies that are highly connected and those that are not.[11] In the United Kingdom (UK), in recognition of the impact of social isolation on health and economic loss, the position of 'Minister of Loneliness' was established in 2018, and the country is taking a progressive approach to the elimination of social isolation.[12] In contrast, Japan, now a super-aged society (more than 21% of the population aged 65 or above),[13] is experiencing a rapid increase in the trend of abstaining from marriage and weakening community and neighbourhood relations,[14] leading to a rise in the number of socially isolated people.[15] In Japan, the proportion of people who rarely or never spend time with those close to them has been reported to be the highest among Organisation for Economic Co-operation and Development countries. In particular, this figure is much higher than in the UK, which has made advances in tackling social isolation (Japan = 15.3%, UK = 5.0%).[16] Owing to differences in social structures and the contexts surrounding social isolation, the impact of social isolation on depression is expected to vary across countries.

Furthermore, the health effects of social isolation may differ depending on the cultural context of social relationships. In East Asian countries, including Japan, there is a familial norm based on the traditional culture of filial piety,[17] which is often contrasted with individualism in Western countries.[18, 19] Based on this cultural

For peer review only

background, Japanese social support networks may be kinship centred, which may be narrower than the types of social networks prevalent in other countries.[20] However, there is a lack of consensus on the health effects of social relationships based on these cultural differences. A previous cross-national study showed that in English men, friendship-based social relationships had a significant impact on longevity, whereas in Japanese men, this impact was associated with family-based social relationships.[21] On the contrary, a study of older adults in the United States (US) and Japan found that while relationships with children were associated with a low level of depression only in Japan, the presence of spouses was important in both countries, but more so in the US.[22] Another comparative study among adults suggested that social contact with friends benefitted women's mental health in the UK but not in Japan.[23] Thus, the family-oriented nature of East Asian societies does not automatically imply the health importance of family-based relationships, and the roles of individual components of social isolation (family, friends, and others) in the mental health of older adults in each country remain controversial.

As the association between social isolation and depression is often described as bidirectional,[24] longitudinal studies are needed to address temporality. However, previous cross-national comparative studies have only employed cross-sectional

For peer review only

designs.[22, 23] Therefore, using longitudinal data from both countries, the present study aims to investigate the association of social isolation with depression onset in England, which has taken advanced measures against social isolation, and Japan, a super-aged society with a rapidly increasing number of socially isolated people.

METHODS

Sample

This longitudinal study was conducted using data from two ongoing prospective cohort studies: the English Longitudinal Study of Ageing (ELSA) and the Japan Gerontological Evaluation Study (JAGES). The ELSA targets independent-living older adults aged ≥ 50, while JAGES participants are community-dwelling individuals aged ≥ 65 who are ineligible for long-term healthcare insurance benefits.[25] Details of the ELSA and JAGES can be found elsewhere.[26, 27] For the present analysis, we used the two waves of data that most closely corresponded with the timing of our study: wave 5 (2010/2011) to wave 6 (2012/2013) for the ELSA, and wave 1 (2010/2011) to wave 2 (2013) for the JAGES. We harmonised the data by including older adults aged ≥ 65, independent in activities of daily living, and without self-reported dementia. For analysis, respondents who scored above the cut-off point for depression on each measure in the respective

For peer review only

cohort at baseline were excluded, and we followed up the onset of depression for two years for the ELSA and 2.5 years for the JAGES.

Depression

Based on a previous cross-national study,[28] depressive symptoms were measured both at baseline and follow-up using eight items from the Center for Epidemiologic Studies Depression Scale (CES-D) in the ELSA[29] and the Geriatric Depression Scale (GDS-15) in the JAGES.[30] To identify possible depressive cases, the CES-D cut-off was ≥ 4 while that for the GDS-15 was ≥ 5.[31, 32] As previously mentioned, respondents with depression at baseline were excluded, and we observed the onset of depression during follow-up.

Social isolation

Social isolation levels were assessed using a modified version of the Social Isolation Index (SII).[33–35] The index was computed with respondents given a point if they: (1) were unmarried or living alone, (2) had poor interaction with children (did not live with their children or had no one to provide emotional or instrumental social support), (3) had poor interaction with relatives (did not have immediate family members providing

For peer review only

emotional or instrumental social support), (4) had poor interaction with friends (less than monthly contact or no friends who could provide emotional or instrumental social support), and (5) had no social participation (no participation in any social or religious groups). The total possible score ranged from 0 to 5, with higher scores indicating greater social isolation. The participants were categorised into the following five groups based on their scores: 0, 1, 2, 3, and 4–5 points. We used the total score and the scores of the five sub-components as predictive variables.

Covariates

The covariates included age, gender, educational attainment, household equivalised income, present illness, self-rated health, smoking, and drinking. Age was categorised as 65–69, 70–74, 75–79, 80–84, and ≥ 85. Based on the ages of respondents who had completed formal education, the age of final educational attainment was categorised as ≤ 15 years, 16–18 years, and ≥ 19 years. Household equivalised income was classified into quintiles. Present illness was classified as 'yes' or 'no' for cancer, heart disease, and stroke. Self-rated health was dichotomised as 'poor' and 'good'. Smoking and drinking were dichotomised as 'never/past' and 'current'.

For peer review only

Statistical analysis

We analysed the ELSA and JAGES data separately because of differences in research design, especially sampling approaches. A longitudinal weight was applied to account for survey non-response for the ELSA but not the JAGES as its design does not allow it. First, we calculated descriptive statistics. Second, we conducted a multivariable logistic regression analysis to examine the association between SII score and depression onset and obtained odds ratios (ORs) and 95% confidence intervals (CIs) for depression onset. Model 1 was not adjusted for covariates while Model 2 was adjusted for all covariates. Additionally, we analysed the association between SII sub-components and depression onset, adjusted for all covariates.

To mitigate potential biases resulting from missing information, we used the multiple imputation approach under the missing at random assumption. We generated 20 imputed datasets for the final analysis, which excluded those who met the exclusion criteria and did not respond to the follow-up surveys, using the multiple imputation by chained equations procedure and pooled the results using Rubin's rule.[36]

The significance level was set at $p < 0.05$. We used R (Version 3.5.2 for Windows) for all statistical analyses.

For peer review only

167 **Ethical considerations**

168 The ELSA investigators received ethical approval for all waves of the study from the
169 National Health Service Research Ethics Committees under the National Research and
170 Ethics Services (MREC/01/2/91). The JAGES protocols were approved by the Ethics
171 Committee on the Research of Human Subjects at Nihon Fukushi University (10-05).

172

173 **Patient and public involvement**

174 No patients were involved in the development of the research question, study design, or
175 data interpretation.

176

177 **RESULTS**

178 A total of 3,331 ELSA respondents and 33,127 JAGES respondents were included in the
179 final analysis. Their baseline characteristics are presented in Table 1. The mean age
180 (standard deviation) was 73.6 (6.9) years for the ELSA and 72.4 (5.4) years for the JAGES.
181 Regarding SII scores, the ELSA had the largest number of respondents with 0 and 1 points,
182 while the JAGES had the largest number with 2 and 3 points. In the ELSA, respondents
183 who were older, male, less educated, had a lower income, had heart disease, had poor
184 self-rated health, smoked, did not consume much alcohol, and had higher baseline

For peer review only

depressive symptom scores had higher SII scores. A similar trend was observed in the JAGES, but here, those who consumed more alcohol had higher SII scores.

For peer review only

Table 1. Respondents' baseline characteristics

	ELSA [a]					JAGES				
	Social Isolation Index score [b]					Social Isolation Index score [b]				
	0	1	2	3	≥ 4	0	1	2	3	≥ 4
	n = 905 (27.2%)	n = 1049 (31.5%)	n = 596 (17.9%)	n = 216 (6.5%)	n = 49 (1.5%)	n = 1402 (4.2%)	n = 5981 (18.0%)	n = 9723 (29.4%)	n = 8735 (26.4%)	n = 2176 (6.6%)
Age (years), (%)										
65–69	41.0	36.4	29.9	27.6	28.3	38.6	40.5	38.0	37.0	35.8
70–74	26.1	28.3	28.8	29.3	23.6	32.1	32.5	31.6	31.0	30.4
75–79	20.9	17.5	19.3	15.8	20.4	19.2	18.3	19.5	20.3	21.9
80–84	9.1	12.2	12.5	14.5	15.5	8.3	6.7	8.3	9.0	8.8
≥ 85	2.8	5.7	9.5	12.8	12.2	1.9	2.0	2.6	2.7	3.1
Gender, (%)										
Men	50.0	46.1	45.3	51.9	65.8	27.2	35.1	47.0	64.4	66.1
Women	50.0	53.9	54.7	48.1	34.2	72.8	64.9	53.0	35.6	33.9
Educational attainment (years), (%)										
≤ 15	44.1	51.3	52.3	60.9	69.6	42.9	38.1	37.9	38.7	48.6
16–18	35.0	33.8	34.6	27.5	14.1	40.9	40.8	39.3	36.8	31.1
≥ 19	17.8	12.5	11.0	10.3	14.0	15.7	20.4	21.8	23.3	18.5
Missing	3.2	2.4	2.1	1.3	2.3	0.5	0.7	0.9	1.2	1.8
Household equivalised income, (%)										
1st quintile (lowest)	11.4	18.3	23.2	31.2	14.4	7.4	10.9	13.9	15.6	21.3
2nd quintile	21.5	23.7	26.1	24.1	29.6	13.2	13.5	15.0	15.5	16.4
3rd quintile	20.9	21.8	18.9	19.5	27.1	22.5	30.6	30.3	30.6	28.0
4th quintile	21.9	19.9	18.1	16.5	16.6	14.3	13.6	12.7	11.3	9.5
5th quintile (highest)	22.7	15.0	12.7	8.3	12.4	31.5	22.5	19.2	16.8	12.9
Missing	1.5	1.3	0.9	0.4	0.0	11.1	8.9	8.9	10.2	11.9
Cancer, (%)										
No	96.5	95.8	97.1	97.5	96.5	91.0	91.2	90.8	90.1	89.7
Yes	3.4	4.2	2.9	2.5	3.5	3.2	3.1	3.1	3.4	3.7
Missing	0.1	0.0	0.0	0.0	0.0	5.8	5.7	6.1	6.5	6.6
Heart disease, (%)										
No	90.7	86.9	88.4	88.3	77.3	91.0	91.2	90.8	90.1	89.7
Yes	9.3	13.0	11.6	11.7	22.7	3.2	3.1	3.1	3.4	3.7
Missing	0.0	0.1	0.0	0.0	0.0	5.8	5.7	6.1	6.5	6.6

For peer review only

For peer review only - http://bmjopen.bmj.com/site/about/guidelines.xhtml

Stroke, (%)										
No	96.3	96.1	97	94.4	96.6	93.3	93.6	93.1	92.5	92.2
Yes	3.7	3.8	3.0	5.6	3.4	0.9	0.8	0.8	1.0	1.2
Missing	0	0.1	0.0	0.0	0.0	5.8	5.7	6.1	6.5	6.6
Self-rated health, (%)										
Good	86.4	79.9	78.8	75.4	71.3	92.0	91.7	90.6	90.2	87.2
Poor	13.6	20.1	21.2	24.6	28.7	7.2	7.7	8.6	9.0	12.2
Missing	0.0	0.0	0.0	0.0	0.0	0.8	0.5	0.8	0.8	0.6
Smoking, (%)										
Never/past	95.5	93.1	89.8	82.9	89.4	86.4	86.1	82.9	81.5	78.4
Current	4.5	6.9	10.2	17.1	10.6	6.4	7.4	9.5	11.3	14.2
Missing	0.0	0.0	0.0	0.0	0.0	7.1	6.5	7.5	7.2	7.3
Drinking, (%)										
Never/past	7.5	11.8	14.9	25.8	17.3	65.0	60.0	56.5	50.4	55.0
Current	91.3	87.0	82.9	72.6	73.6	30.2	35.7	38.6	44.7	40.4
Missing	1.3	1.2	2.2	1.6	9.2	4.8	4.3	4.9	4.9	4.6
CES-D score at baseline, (%)										
0	57.2	52.0	45.5	42.2	54.0					
1	27.3	26.2	28.6	31.8	25.5					
2	9.8	13.8	17.1	11.9	15.1					
3	5.7	8.0	8.8	14.2	5.4					
GDS score at baseline, (%)										
0						20.5	30.4	29.0	26.4	24.3
1						25.5	28.4	28.6	26.9	27.0
2						22.5	20.0	19.8	21.5	21.1
3						17.5	13.1	13.5	14.7	15.9
4						14.1	8.1	9.1	10.5	11.7

ELSA, English Longitudinal Study of Ageing; JAGES, Japan Gerontological Evaluation Study; CES-D, Center for Epidemiologic Studies Depression Scale; GDS, Geriatric Depression Scale

[a] ELSA data after sampling weight

[b] Missing data: ELSA, n = 516; JAGES, n = 5,110

For peer review only

Table 2 presents the description of social isolation and depression onset. At follow-up, 201 (6.0%) ELSA respondents and 4,456 (13.5%) JAGES respondents exhibited depression onset. In both studies, higher SII scores were associated with an increased risk of depression onset. Regarding SII sub-components, ELSA respondents who were unmarried or living alone were more likely to have depression, while this was the case with JAGES respondents with no social participation.

For peer review only

199 **Table 2. Description of social isolation status and depression onset**

		ELSA [a]		JAGES	
		CES-D score at follow-up		GDS score at follow-up	
		< 4	≥ 4	< 5	≥ 5
		n = 3130 (94.0%)	n = 201 (6.0%)	n = 28671 (86.5%)	n = 4456 (13.5%)
Social Isolation Index score, (%)					
0		27.5	13.5	4.4	3.0
1		31.2	29.3	18.6	14.3
2		17.9	18.9	29.8	26.3
3		6.5	10.1	26.2	27.5
≥ 4		1.5	2.9	6.1	9.3
Missing		15.4	25.3	14.8	19.7
Social Isolation Index sub-components, (%)					
Unmarried or living alone	No	71.9	58.5	88.3	85.2
	Yes	28.1	41.5	10.2	12.7
	Missing	0.0	0.0	1.4	2.1
Poor interaction with children	No	81.8	73.4	26.6	25.0
	Yes	12.5	14.7	71.7	72.9
	Missing	5.7	11.8	1.7	2.1
Poor interaction with relatives	No	76.3	68.1	41.1	38.7
	Yes	17.3	18.6	54.4	56.0
	Missing	6.4	13.4	4.5	5.4
Poor interaction with friends	No	72.2	66.0	37.1	31.2
	Yes	18.4	21.6	58.2	63.8
	Missing	9.4	12.4	4.6	5.0
No social participation	No	61.7	46.3	75.0	63.9
	Yes	28.4	32.5	13.0	20.0
	Missing	10.0	21.2	11.7	16.1

200 ELSA, English Longitudinal Study of Ageing; JAGES, Japan Gerontological Evaluation
201 Study; CES-D, Center for Epidemiologic Studies Depression Scale; GDS, Geriatric
202 Depression Scale

203 [a] ELSA data after sampling weight

For peer review only

204 Table 3 depicts the association between SII scores and depression onset.
205 Multivariable analysis showed that higher SII scores were associated with a higher risk
206 of depression onset in both studies after adjusting for all covariates. In the ELSA, the OR
207 of depression onset was significantly higher from a score ≥ one point (OR [95% CI]
208 compared with zero points, one: 1.68 [1.02–2.75], two: 1.77 [1.03–3.05], three: 2.64
209 [1.37–5.12], ≥ four: 4.01 [1.43–11.22], *p* for trend = 0.015). In the JAGES, as SII scores
210 increased, the OR of depression onset gradually increased, reaching significance at ≥
211 three points (OR [95% CI] compared with zero points, one: 1.10 [0.89–1.35], two: 1.15
212 [0.94–1.40], three: 1.28 [1.04–1.56], ≥ four: 1.48 [1.18–1.85], *p* for trend < 0.001). These
213 results showed almost the same tendency as the complete case analysis without multiple
214 imputation (Supplementary Table 1).

For peer review only

Table 3. Association between social isolation and depression onset: multivariable logistic regression analysis

	ELSA		JAGES	
	Crude OR (95% CI)	Adjusted OR (95% CI) [a]	Crude OR (95% CI)	Adjusted OR (95% CI) [a]
Social Isolation Index score				
0	1.00 (reference)	1.00 (reference)	1.00 (reference)	1.00 (reference)
1	1.92** (1.19–3.10)	1.68* (1.02–2.75)	1.14 (0.94–1.39)	1.10 (0.89–1.35)
2	2.15** (1.28–3.62)	1.77* (1.03–3.05)	1.32** (1.09–1.60)	1.15 (0.94–1.40)
3	3.19*** (1.73–5.90)	2.64** (1.37–5.12)	1.57*** (1.30–1.90)	1.28* (1.04–1.56)
≥ 4	3.85** (1.46–10.18)	4.01** (1.43–11.22)	2.26*** (1.83–2.79)	1.48*** (1.18–1.85)
	p for trend < 0.001	p for trend = 0.015	p for trend < 0.001	p for trend < 0.001

* $p < 0.05$; ** $p < 0.01$; *** $p < 0.001$

ELSA, English Longitudinal Study of Ageing; JAGES, Japan Gerontological Evaluation Study; CI, confidence interval; OR, odds ratio

[a] Adjusted for age, gender, educational attainment, household equivalised income, present illness (cancer, heart disease, and stroke), self-rated health, smoking, drinking, and depression score at baseline (Center for Epidemiologic Studies Depression Scale for the ELSA and Geriatric Depression Scale for the JAGES).

For peer review only - http://bmjopen.bmj.com/site/about/guidelines.xhtml

Table 4 presents the associations of SII sub-components with depression onset. In the ELSA, sub-components were not significant, although poor interaction with children was marginally significant (OR [95% CI], with 'none' as the reference; unmarried or living alone: 1.13 [0.80–1.60], poor interaction with children: 1.55 [1.00–2.41], poor interaction with relatives: 1.24 [0.79–1.94], poor interaction with friends: 1.15 [0.77–1.71], no social participation: 1.22 [0.80–1.87]). In the JAGES, poor interaction with children and no social participation were significantly associated with depression onset after adjusting for all covariates (OR [95% CI], with 'none' as the reference; unmarried or living alone: 1.11 [1.00–1.24], poor interaction with children: 1.09 [1.01–1.19], poor interaction with relatives: 1.04 [0.96–1.12], poor interaction with friends: 1.03 [0.95–1.11], no social participation: 1.28 [1.17–1.40]). These results were similar to those obtained from the complete case analysis (Supplementary Table 2).

For peer review only

Table 4. Association between sub-components of social isolation and depression onset: multivariable logistic regression analysis

	ELSA	JAGES
	Adjusted OR (95% CI) [a]	Adjusted OR (95% CI) [a]
Social Isolation Index sub-components (reference: none)		
Unmarried or living alone	1.13 (0.80–1.60)	1.11† (1.00–1.24)
Poor interaction with children	1.55† (1.00–2.41)	1.09* (1.01–1.19)
Poor interaction with relatives	1.24 (0.79–1.94)	1.04 (0.96–1.12)
Poor interaction with friends	1.15 (0.77–1.71)	1.03 (0.95–1.11)
No social participation	1.22 (0.80–1.87)	1.28*** (1.17–1.40)

* $p < 0.05$; ** $p < 0.01$; *** $p < 0.001$; † $p < 0.1$

ELSA, English Longitudinal Study of Ageing; JAGES, Japan Gerontological Evaluation Study; CI, confidence interval; OR, odds ratio

[a] Adjusted for age, gender, educational attainment, equivalent income, present illness (cancer, heart disease, and stroke), self-rated health, smoking, drinking, and depression score at baseline (Center for Epidemiologic Studies Depression Scale for the ELSA and Geriatric Depression Scale for the JAGES).

For peer review only

DISCUSSION

To the best of our knowledge, this is the first cross-national longitudinal study of the association of social isolation with depression among older English and Japanese adults. Social isolation was significantly associated with depression onset in both countries. Our results support previous longitudinal findings on social relationships and mental health in older adults in England[37] and Japan.[38] Using data frames that were similar with regard to assessment and covariates, we showed that social isolation is a common risk factor for depression in England and Japan despite country-specific cultural differences regarding social relationships. Thus, our results suggest that to safeguard the mental health of older adults, addressing social isolation is a global need.

The association between social isolation and depression was somewhat stronger in England than in Japan. These results are similar to a previous report concerning mortality among older adults in England and Japan.[11] Although we cannot make direct comparisons because of variations in cohort follow-up periods and depression measurement, there are several possible reasons for this pattern of findings. The impact of social factors could be different depending on the group and society to which one belongs. This is best understood in the context of the concept of relative deprivation.[39] In other words, higher levels of relative social isolation may induce greater psychological

For peer review only

stress. A previous study showed that rich community ties and cohesion were protective factors for health but could have a negative effect on those who were not socially involved.[40] Being isolated in a connected society such as the UK may represent a more severe condition, with a stronger negative impact on mental health.

Our results showed that poor interaction with children was significant with regard to depression onset in Japan. In England, while the association was marginal, of the components of social isolation, poor interaction with children had the greatest effect. The lack of interaction with children could have an adverse effect on the mental health of older adults in both countries. Previous studies in England[41] and Japan[22] have reported that social support from children can contribute to alleviating depression, and our results point in the same direction. Older adults without children can be considered a vulnerable group, because adult children, in particular, are often the main source of positive social support for older parents.[42] Older parents have certain expectations with regard to receiving support from their children, and situations wherein these expectations are not met may lead to depressive mood.[43] On the contrary, a previous study reported no association between the presence of children and depression among older adults in the US.[22] Owing to strong spousal relationships in the US, the effect of the presence of children might be relatively small. Thus, our study confirmed the adverse effects of poor

For peer review only

interaction with children common to England and Japan, but international generalizability can only be established based on further research considering the cultural background of family relationships in individual countries.

Although traditionally Japan is a country where adult children are expected to demonstrate reciprocity with their parents owing to the strong family and kinship-based cultural background,[44] in this study, the effect of interaction with children on depression was relatively modest. In recent years, with trends such as adult children commonly living apart from their parents after marriage[45] and the development of public long-term care services for the ageing population,[46] Japan's family system has not remained as traditional as before. Therefore, interaction with children may not be as essential to the health of older adults as before. However, despite these cultural transitions, we believe that interaction with children has some value with regard to preventing depression in old age in Japan.

Social participation was a strong protective factor for depression onset in Japan, whereas there was no association in England, although the OR was somewhat greater. Several previous studies have reported that social participation helps prevent depression onset.[37, 47–49] Our results pertaining to Japan support these reports. On the contrary, the protective effects of social participation on mental health have been shown to vary

For peer review only

depending on the type of organisation an individual is involved with,[48] the individual's attitude towards participation,[48] and the duration[37] and frequency[49] of participation. Regarding the present study, in the English context, the role of social participation in depression prevention might have been unidentifiable because of differences in the effects of these participation contexts. We only took into account social participation, without delving into specific types. Thus, the context of effective social participation, such as type, duration, and role in the organisation in both countries, requires further investigation. Despite these challenges, our findings suggest that in Japan, social isolation prevention measures based on the promotion of social participation could be beneficial for safeguarding the mental health of older adults.

This study has several strengths. First, it is the first cross-national population-level investigation of the association of social isolation with depression onset using a unified data frame. Second, by using two longitudinal datasets, we were able to examine the prospective association between social isolation and depression. Third, the use of large-scale data allowed us to detect the effects of relatively rare situations of severe social isolation.

However, certain limitations cannot be ignored. First, the measurement of depression in the two cohorts was not the same. Therefore, we could not directly compare

For peer review only

311 depression onset in the two countries. However, these measurements were also used in a
312 previous cross-national comparison study in England and Japan,[28] and we were able to
313 examine the association between social isolation and depression onset in both countries
314 using the same data frame. Second, we used social support for the assessment of social
315 contact for some items in order to be able to use the same SII. Therefore, cultural
316 differences in expectations regarding the receipt of social support in both countries might
317 have caused information biases. For instance, expectations regarding social support from
318 relatives could originally have been higher in Japan,[44] leading to overestimation of
319 social isolation levels. Third, regarding the items of the SII, the questions and their
320 response options in the ELSA and JAGES were not exactly the same, nor were they
321 strictly authorised through procedures such as reverse translation and confirming
322 reliability and validity. However, we believe it is certainly meaningful to evaluate social
323 isolation using the same framework. Finally, there were differences in study design in the
324 data from the two cohorts, such as sampling method and follow-up period. We, therefore,
325 made efforts to harmonise the data: those aged ≤ 64, with dementia, and dependent in
326 activities of daily living were excluded from the analysis. Moreover, the ELSA presents
327 nationally representative population data, while the JAGES does not. However, the
328 JAGES sample is representative of areas from a nationwide ageing study in which about

For peer review only

one-fifth of all prefectures (nine out of 47) were enrolled. Even so, unlike the ELSA, analysis in the JAGES does not use sampling weights, which may lead to selection bias.

CONCLUSION

We examined the association between social isolation and depression onset among older adults in England and Japan, which have different cultural contexts regarding social relationships, finding a significant association in both countries. Thus, globally, tackling social isolation must be prioritised to safeguard the mental health of older adults. Particularly in Japan, the promotion of interaction with children and social participation could be key factors in addressing social isolation.

ACKNOWLEDGEMENTS

We wish to express our deepest gratitude to the members in the Japan Gerontological Evaluation Study. We would also like to thank everyone who participated in the surveys.

AUTHORS' CONTRIBUTIONS

All authors contributed to the conception and design of this study. Data collection was primarily conducted by MS, JA, NC, KO, and KK. Analyses were performed by TN, MS,

For peer review only

JA, TT, SK, and TI. TN prepared the initial manuscript and MS, JA, NC, TT, SK, TI, KO, and KK significantly contributed to revising it. All authors read and approved the final manuscript.

FUNDING STATEMENT

This work was supported by a grant from the Grants-in-Aid for Scientific Research (18KK0057, 19K24277) from the Japan Society for the Promotion of Science. The English Longitudinal Study of Ageing was developed by a team of researchers based at the University College London, NatCen Social Research, and the Institute for Fiscal Studies. The data were collected by NatCen Social Research. The funding is currently provided by the National Institute of Aging (R01AG017644) and a consortium of UK government departments coordinated by the National Institute for Health Research. The Japan Gerontological Evaluation Study is supported by the Ministry of Education, Culture, Sports, Science and Technology-Japan-Supported Program for the Strategic Research Foundation at Private Universities (2009-2013), Japan Society for the Promotion of Science KAKENHI Grant Numbers (JP18390200, JP22330172, JP22390400, JP23243070, JP23590786, JP23790710, JP24390469, JP24530698, JP24683018, JP25253052, JP25870573, JP25870881, JP26285138, JP26882010, JP15H01972),

For peer review only

Health Labour Sciences Research Grants (H22-Choju-Shitei-008, H24-Junkanki -Ippan-007, H24-Chikyukibo-Ippan-009, H24-Choju-Wakate-009, H25-Kenki-Wakate-015, H25-Choju-Ippan-003, H26-Irryo-Shitei-003 [Fukkou], H26-Choju-Ippan-006, H27-Ninchisyou-Ippan-001, H28-chojuIppan-002, H28-Ninchisho-Ippan-002, H30-Kenki-Ippan-006, H30-Junkankitou-Ippan-004), Japan Agency for Medical Research and Development (JP17dk0110017, JP18dk0110027, JP18ls0110002, JP18le0110009, JP19dk0110034), Research Funding for Longevity Sciences from the National Center for Geriatrics and Gerontology (24-17, 24-23, 29-42, 30-22, 20-40), Open Innovation Platform with Enterprises, Research Institute and Academia (OPERA, JPMJOP1831) from the Japan Science and Technology Agency, and a Research Grant from the Health Science Center Foundation (2019-2020). The funding sources had no role in the study design, data collection and analysis, decision to publish or preparation of the manuscript.

DATA SHARING STATEMENT

For the JAGES, all enquiries are to be addressed to the data management committee via e-mail: dataadmin.ml@jages.net. All JAGES datasets have ethical or legal restrictions for public deposition because of the inclusion of sensitive information about the human participants. Regarding the ELSA, data are available in an open-access repository at

For peer review only

http://www. dataarchive.ac.uk/.

COMPETING INTERESTS STATEMENT

The authors declare no conflict of interest.

REFERENCES

1. Townsend P. The family life of old people: an investigation in East London. *Sociol Rev* 1955;3:175–95. doi: 10.1111/j.1467-954X.1955.tb01052.x

2. Holt-Lunstad J, Smith TB, Layton JB. Social relationships and mortality risk: a meta-analytic review. *PLoS Med* 2010;7:e1000316. doi:10.1371/journal.pmed.1000316

3. Valtorta NK, Kanaan M, Gilbody S, et al. Loneliness and social isolation as risk factors for coronary heart disease and stroke: systematic review and meta-analysis of longitudinal observational studies. *Heart* 2016;102:1009–16. doi:10.1136/heartjnl-2015-308790

4. Livingston G, Sommerlad A, Orgeta V, et al. Dementia prevention, intervention, and care. *Lancet* 2017;390:2673–734. doi:10.1016/s0140-6736(17)31363-6

5. Leigh-Hunt N, Bagguley D, Bash K, et al. An overview of systematic reviews on the public health consequences of social isolation and loneliness. *Public Health*

For peer review only

2017;152:157–71. doi:10.1016/j.puhe.2017.07.035

6. Santini ZI, Koyanagi A, Tyrovolas S, et al. The association between social relationships and depression: a systematic review. *J Affect Disord* 2015;175:53–65. doi:10.1016/j.jad.2014.12.049

7. Kawachi I, Berkman LF. Social ties and mental health. *J Urban Health* 2001;78:458–67. doi:10.1093/jurban/78.3.458

8. Sivertsen H, Bjorklof GH, Engedal K, et al. Depression and quality of life in older persons: a review. *Dement Geriatr Cogn Disord* 2015;40:311–39. doi:10.1159/000437299

9. Chen CM, Mullan J, Su YY, et al. The longitudinal relationship between depressive symptoms and disability for older adults: a population-based study. *J Gerontol A Biol Sci Med Sci* 2012;67:1059–67. doi:10.1093/gerona/gls074

10. World Federation for Mental Health. Depression: a global crisis. World Mental Health Day, October 10 2012. 2012. https://www.who.int/mental_health/management/depression/wfmh_paper_depression_wmhd_2012.pdf (accessed 7 Feb 2020).

11. Saito M, Aida J, Cable N, et al. Cross-national comparison of social isolation and mortality among older adults: a 10-year follow-up study in Japan and England.

For peer review only

Geriatr Gerontol Int 2020 doi: 10.1111/ggi.14118

12. HM Government. A connected society: a strategy for tackling loneliness – laying the foundations for change. 2018. https://assets.publishing.service.gov.uk/government/uploads/system/uploads/attachment_data/file/750909/6.4882_DCMS_Loneliness_Strategy_web_Update.pdf (accessed 12 Oct 2020).

13. Muramatsu N, Akiyama H. Japan: super-aging society preparing for the future. *Gerontologist* 2011;51:425–32. doi: 10.1093/geront/gnr067

14. National Institute of Population and Social Security Research. Household projections for Japan. http://www.ipss.go.jp/pp-ajsetai/e/hprj_98/hprj_98.html (accessed 12 Oct 2020).

15. Murayama H, Shibui Y, Fukuda Y, et al. A new crisis in Japan-social isolation in old age. *J Am Geriatr Soc* 2011;59:2160–2. doi:10.1111/j.1532-5415.2011.03640.x

16. Organisation for Economic Co-operation and Development. Society at a Glance: OECD Social Indicators. Paris: OECD Publishing 2005.

17. Takagi E, Silverstein M. Intergenerational coresidence of the Japanese elderly:are cultural norms proactive or reactive? *Research on Aging* 2006;28:473–92. doi: 10.1177/0164027506287788

For peer review only

18. Knight B, Robinson Shurgot G, Longmire C, et al. Cross cultural issues in caregiving for persons with dementia: do familism values reduce burden and distress? *Aging International* 2002;27:70–94. doi: 10.1007/s12126-003-1003-y

19. Knight BG, Sayegh P. Cultural values and caregiving: the updated sociocultural stress and coping model. *J Gerontol B Psychol Sci Soc Sci* 2009;65B(1):5–13. doi: 10.1093/geronb/gbp096 %

20. Cabinet Office. International Comparative Survey on the Life and Awareness of the Elderly in 2015. Cabinet Office, Tokyo. 2015. https://www8.cao.go.jp/kourei/ishiki/h27/zentai/index.html (accessed 30 Jan 2021).

21. Aida J, Cable N, Zaninotto P, et al. Social and behavioural determinants of the difference in survival among older adults in Japan and England. *Gerontology* 2018;64:266–77. doi:10.1159/000485797

22. Sugisawa H, Shibata H, Hougham GW, et al. The impact of social ties on depressive symptoms in U.S. and Japanese elderly. *J Soc Issues* 2003;58:785–804. doi:10.1111/1540-4560.00290

23. Cable N, Chandola T, Lallukka T, et al. Country specific associations between social contact and mental health: evidence from civil servant studies across Great Britain,

For peer review only

Japan and Finland. *Public Health* 2016;137:139–46. doi: 10.1016/j.puhe.2015.10.013

24. Santini ZI, Jose PE, York Cornwell E, et al. Social disconnectedness, perceived isolation, and symptoms of depression and anxiety among older Americans (NSHAP): a longitudinal mediation analysis. *Lancet Public Health* 2020;5:e62–70. doi:10.1016/s2468-2667(19)30230-0

25. Mossialos E, Djordjevic A, Osborn R, et al. International profiles of health care systems. 2017. https://www.commonwealthfund.org/sites/default/files/documents/___media_files_publications_fund_report_2017_may_mossialos_intl_profiles_v5.pdf (accessed 12 Oct 2020).

26. Kondo K. Progress in aging epidemiology in Japan: the JAGES Project. *J Epidemiol* 2016;26:331–6. doi:10.2188/jea.JE20160093

27. Steptoe A, Breeze E, Banks J, et al. Cohort profile: the English longitudinal study of ageing. *Int J Epidemiol* 2013;42:1640–8. doi: 10.1093/ije/dys168

28. Cable N, Chandola T, Aida J, et al. Can sleep disturbance influence changes in mental health status? Longitudinal research evidence from ageing studies in England and Japan. *Sleep Med* 2017;30:216–21. doi:10.1016/j.sleep.2016.11.017

For peer review only

29. Marmot M, Banks J, Blundell R, et al. Health, Wealth and Lifestyles of the Older Population in England: The 2002 English Longitudinal Study of Ageing. London: Institute for Fiscal Studies 2003.

30. Wada T, Ishine M, Kita T, et al. Depression screening of elderly community-dwelling Japanese. *J Am Geriatr Soc* 2003;51:1328–9. doi: 10.1046/j.1532-5415.2003.514207.x

31. Gallagher D, Kiss A, Lanctot K, et al. Depressive symptoms and cognitive decline: a longitudinal analysis of potentially modifiable risk factors in community dwelling older adults. *J Affect Disord* 2016;190:235–40. doi:10.1016/j.jad.2015.09.046

32. Burke WJ, Roccaforte WH, Wengel SP. The short form of the Geriatric Depression Scale: a comparison with the 30-item form. *J Geriatr Psychiatry Neurol* 1991;4:173–8. doi:10.1177/089198879100400310

33. Ikeda T, Cable N, Saito M, et al. Association between social isolation and smoking in Japan and England. *J Epidemiol* 2020 doi: 10.2188/jea.JE20200138

34. Tsuji T, Saito M, Ikeda T, et al. Change in the prevalence of social isolation among the older population from 2010 to 2016: a repeated cross-sectional comparative study of Japan and England. *Arch Gerontol Geriatr* 2020;91:104237. doi: 10.1016/j.archger.2020.104237

For peer review only

35. Shankar A, McMunn A, Banks J, et al. Loneliness, social isolation, and behavioral and biological health indicators in older adults. *Health Psychol* 2011;30:377–85. doi:10.1037/a0022826

36. White IR, Royston P, Wood AM. Multiple imputation using chained equations: issues and guidance for practice. *Stat Med* 2011;30:377–99. doi:10.1002/sim.4067

37. Cruwys T, Dingle GA, Haslam C, et al. Social group memberships protect against future depression, alleviate depression symptoms and prevent depression relapse. *Soc Sci Med* 2013;98:179–86. doi:10.1016/j.socscimed.2013.09.013

38. Koizumi Y, Awata S, Kuriyama S, et al. Association between social support and depression status in the elderly: results of a 1-year community-based prospective cohort study in Japan. *Psychiatry Clin Neurosci* 2005;59:563–9. doi:10.1111/j.1440-1819.2005.01415.x

39. Smith HJ, Pettigrew TF, Pippin GM, et al. Relative deprivation: a theoretical and meta-analytic review. *Pers Soc Psychol Rev* 2012;16:203–32. doi:10.1177/1088868311430825

40. Amemiya A, Saito J, Saito M, et al. Social capital and the improvement in functional ability among older people in Japan: a multilevel survival analysis using JAGES data. *Int J Environ Res Public Health* 2019;16:1310. doi:10.3390/ijerph16081310

For peer review only

41. Stafford M, McMunn A, Zaninotto P, et al. Positive and negative exchanges in social relationships as predictors of depression: evidence from the English Longitudinal Study of Aging. *J Aging Health* 2011;23:607–28. doi:10.1177/0898264310392992

42. Koropeckyj-Cox T. Loneliness and depression in middle and old age: are the childless more vulnerable? *J Gerontol B Psychol Sci Soc Sci* 1998;53:S303–12. doi: 10.1093/geronb/53b.6.s303

43. Lee GR, Netzer JK, Coward RT. Depression among older parents: the role of intergenerational exchange. *J Marriage Family* 1995;57:823–33. doi: 10.2307/353935

44. Brown JW, Liang J, Krause N, et al. Transitions in living arrangements among elders in Japan: does health make a difference? *J Gerontol B Psychol Sci Soc Sci* 2002;57:S209–20. doi: 10.1093/geronb/57.4.s209

45. Takagi E, Silverstein M. Purchasing piety? Coresidence of married children with their older parents in Japan. *Demography* 2011;48:1559–79. doi:10.1007/s13524-011-0053-0

46. Tsutsui T, Muramatsu N. Care-needs certification in the long-term care insurance system of Japan. *J Am Geriatr Soc* 2005;53(3):522–7. doi: 10.1111/j.1532-

For peer review only

5415.2005.53175.x

47. Takagi D, Kondo K, Kawachi I. Social participation and mental health: moderating effects of gender, social role and rurality. *BMC Public Health* 2013;13:701. doi:10.1186/1471-2458-13-701

48. Tomioka K, Kurumatani N, Hosoi H. Positive and negative influences of social participation on physical and mental health among community-dwelling elderly aged 65-70 years: a cross-sectional study in Japan. *BMC Geriatr* 2017;17:111. doi: 10.1186/s12877-017-0502-8

49. Choi E, Han KM, Chang J, et al. Social participation and depressive symptoms in community-dwelling older adults: emotional social support as a mediator. *J Psychiatr Res* 2020. doi: 10.1016/j.jpsychires.2020.10.043

For peer review only

Supplementary Table 1. Association between social isolation and depression onset, by complete case analysis

		ELSA		JAGES	
		Crude OR (95% CI)	Adjusted OR (95% CI)	Crude OR (95% CI)	Adjusted OR (95% CI)
Social Isolation Index score	0	1.00 (reference)	1.00 (reference)	1.00 (reference)	1.00 (reference)
	1	1.92** (1.19–3.10)	1.56† (0.94–2.60)	1.14 (0.94–1.39)	1.11 (0.87–1.41)
	2	2.15** (1.28–3.62)	1.71† (0.98–2.99)	1.32** (1.09–1.60)	1.20 (0.95–1.51)
	3	3.19*** (1.73–5.90)	2.50** (1.27–4.90)	1.57*** (1.30–1.90)	1.30* (1.03–1.64)
	≥ 4	3.85** (1.46–10.18)	5.17** (1.83–14.66)	2.26*** (1.83–2.79)	1.47** (1.13–1.91)
		p for trend < 0.001	*p* for trend < 0.001	*p* for trend < 0.001	*p* for trend < 0.001
Age (years)	65–69		1.00 (reference)		1.00 (reference)
	70–74		1.37 (0.83–2.25)		1.16** (1.05–1.30)
	75–79		1.24 (0.71–2.14)		1.31*** (1.16–1.48)
	80–84		1.66† (0.92–2.99)		1.60*** (1.37–1.88)
	≥ 85		0.97 (0.45–2.09)		1.79*** (1.39–2.30)
Gender	Male		1.00 (reference)		1.00 (reference)
	Female		1.58* (1.07–2.34)		1.00 (0.90–1.11)
Educational attainment (years)	< 15		1.00 (reference)		1.00 (reference)
	16–18		1.05 (0.68–1.60)		0.81*** (0.74–0.89)
	≥ 19		1.30 (0.70–2.41)		0.68*** (0.60–0.78)
Household equivalised income	1st quintile (lowest)		1.00 (reference)		1.00 (reference)
	2nd quintile		0.70 (0.40–0.21)		0.95 (0.83–1.09)
	3rd quintile		1.11 (0.66–1.87)		0.76*** (0.67–0.87)
	4th quintile		0.82 (0.45–1.50)		0.67*** (0.57–0.79)
	5th quintile (highest)		0.84 (0.42–1.68)		0.65*** (0.56–0.76)

For peer review only

Cancer	No	1.00 (reference)	1.00 (reference)
	Yes	0.95 (0.32–2.81)	1.16 (0.93–1.43)
Heart disease	No	1.00 (reference)	1.00 (reference)
	Yes	1.13 (0.67–1.92)	1.20 (1.05–1.37)
Stroke	No	1.00 (reference)	1.00 (reference)
	Yes	1.3 (0.56–3.02)	1.18 (0.80–1.73)
Self-rated health	Good	1.00 (reference)	1.00 (reference)
	Poor	1.71* (1.13–2.59)	1.63*** (1.44–1.85)
CES-D score at baseline		1.90*** (1.60–2.25)	
GDS score at baseline			1.92*** (1.86–1.99)
Smoking	Never/past	1.00 (reference)	1.00 (reference)
	Current	0.76 (0.76–1.56)	1.29*** (1.12–1.48)
Drinking	Never/past	1.00 (reference)	1.00 (reference)
	Current	1.41 (0.81 – 2.44)	1.02 (0.93 – 1.13)

*, $p < 0.05$; **, $p < 0.01$; ***, $p < 0.001$; †, $p < 0.1$

ELSA, English Longitudinal Study of Ageing; JAGES, Japan Gerontological Evaluation Study; CES-D, Center for Epidemiologic Studies Depression Scale; CI, confidence interval; GDS, Geriatric Depression Scale; OR, odds ratio

For peer review only

Supplementary Table 2. Association between sub-components of social isolation and depression onset, by complete case analysis

		ELSA		JAGES	
		Crude OR (95% CI)	Adjusted OR (95% CI)	Crude OR (95% CI)	Adjusted OR (95% CI)
Social Isolation Index sub-components (ref; none)					
Unmarried or living alone		1.91*** (1.34–2.72)	1.27 (0.84–1.93)	1.33*** (1.18–1.49)	1.02 (0.88–1.19)
Poor interaction with children		1.08 (0.68–1.74)	1.51 (0.91–2.50)	1.01 (0.98–1.10)	1.13* (1.02–1.25)
Poor interaction with relatives		1.25 (0.83–1.90)	1.33 (0.85–2.08)	1.05 (0.97–1.13)	0.98 (0.90–1.08)
Poor interaction with friends		1.19 (0.78–1.80)	1.21 (0.78–1.87)	1.23 (1.14–1.33)	1.11* (1.01–1.22)
No social participation		1.54* (1.08–2.19)	1.46† (0.98–2.16)	1.70 (1.56–1.86)	1.22*** (1.09–1.36)
Age (years)	65–69		1.00 (reference)		1.00 (reference)
	70–74		1.37 (0.83–2.25)		1.17** (1.05–1.30)
	75–79		1.25 (0.72–2.17)		1.31*** (1.16–1.49)
	80–84		1.68† (0.92–3.04)		1.60*** (1.36–1.88)
	≥ 85		1.00 (0.46–2.21)		1.78*** (1.38–2.30)
Gender	Male		1.00 (reference)		1.00 (reference)
	Female		1.59* (1.07–2.37)		1.00 (0.89–1.11)
Educational attainment (years)	< 15		1.00 (reference)		1.00 (reference)
	10–12		1.04 (0.67–1.60)		0.81*** (0.74–0.90)
	≥ 19		1.31 (0.70–2.46)		0.69*** (0.61–0.78)
Household equivalised income	1st quintile (lowest)		1.00 (reference)		1.00 (reference)
	2nd quintile		0.71 (0.41–1.23)		0.95 (0.83–1.09)
	3rd quintile		1.13 (0.67–1.93)		0.76*** (0.67–0.86)
	4th quintile		0.83 (0.45–1.52)		0.67*** (0.57–0.79)
	5th quintile (highest)		0.85 (0.42–1.71)		0.66*** (0.57–0.76)
Cancer	No		1.00 (reference)		1.00 (reference)
	Yes		0.95 (0.32–2.81)		1.15 (0.93–1.43)

For peer review only

Heart disease	No	1.00 (reference)	1.00 (reference)
	Yes	1.16 (0.69–1.96)	1.20^{**} (1.05–1.37)
Stroke	No	1.00 (reference)	1.00 (reference)
	Yes	1.28 (0.55–2.99)	1.18 (0.80–1.73)
Self-rated health	Good	1.00 (reference)	1.00 (reference)
	Poor	1.71^{*} (1.13–2.58)	1.62^{***} (1.43–1.83)
CES-D score at baseline		1.89^{***} (1.59–2.25)	1.00 (reference)
GDS score at baseline			1.92^{***} (1.85–1.99)
Smoking	Never/past	1.00 (reference)	1.00 (reference)
	Current	0.74 (0.36–1.53)	1.29^{***} (1.13–1.48)
Drinking	Never/past	1.00 (reference)	1.00 (reference)
	Current	1.39 (0.80–2.40)	1.03 (0.93–1.14)

*, $p < 0.05$; **, $p < 0.01$; ***, $p < 0.001$; †, $p < 0.1$

ELSA, English Longitudinal Study of Ageing; JAGES, Japan Gerontological Evaluation Study; CES-D, Center for Epidemiologic Studies Depression Scale; CI, confidence interval; GDS, Geriatric Depression Scale; OR, odds ratio

For peer review only

STROBE Statement—Checklist of items that should be included in reports of ***cohort studies***

	Item No	Recommendation	Page No
**Title and abstract**	1	(*a*) Indicate the study's design with a commonly used term in the title or the abstract	1, 3
		(*b*) Provide in the abstract an informative and balanced summary of what was done and what was found	3-4
**Introduction**			
Background/rationale	2	Explain the scientific background and rationale for the investigation being reported	6-9
Objectives	3	State specific objectives, including any prespecified hypotheses	9
**Methods**			
Study design	4	Present key elements of study design early in the paper	9
Setting	5	Describe the setting, locations, and relevant dates, including periods of recruitment, exposure, follow-up, and data collection	9-10
Participants	6	(*a*) Give the eligibility criteria, and the sources and methods of selection of participants. Describe methods of follow-up	9-10
		(*b*) For matched studies, give matching criteria and number of exposed and unexposed	Not applicable
Variables	7	Clearly define all outcomes, exposures, predictors, potential confounders, and effect modifiers. Give diagnostic criteria, if applicable	10-11
Data sources/ measurement	8*	For each variable of interest, give sources of data and details of methods of assessment (measurement). Describe comparability of assessment methods if there is more than one group	10-11
Bias	9	Describe any efforts to address potential sources of bias	12
Study size	10	Explain how the study size was arrived at	Not applicable
Quantitative variables	11	Explain how quantitative variables were handled in the analyses. If applicable, describe which groupings were chosen and why	10-11
Statistical methods	12	(*a*) Describe all statistical methods, including those used to control for confounding	11-12
		(*b*) Describe any methods used to examine subgroups and interactions	Not applicable
		(*c*) Explain how missing data were addressed	12
		(*d*) If applicable, explain how loss to follow-up was addressed	Not applicable
		(*e*) Describe any sensitivity analyses	Not applicable
**Results**			
Participants	13*	(a) Report numbers of individuals at each stage of study—eg numbers potentially eligible, examined for eligibility, confirmed eligible, included in the study, completing follow-up, and analysed	13
		(b) Give reasons for non-participation at each stage	Not applicable
		(c) Consider use of a flow diagram	Not applicable
Descriptive data	14*	(a) Give characteristics of study participants (eg demographic, clinical, social) and information on exposures and potential confounders	13-14
		(b) Indicate number of participants with missing data for each variable of interest	15-16
		(c) Summarise follow-up time (eg, average and total amount)	10
Outcome data	15*	Report numbers of outcome events or summary measures over time	17-18

For peer review only

Main results	16	(*a*) Give unadjusted estimates and, if applicable, confounder-adjusted estimates and their precision (eg, 95% confidence interval). Make clear which confounders were adjusted for and why they were included	19-21
		(*b*) Report category boundaries when continuous variables were categorized	Not applicable
		(*c*) If relevant, consider translating estimates of relative risk into absolute risk for a meaningful time period	Not applicable
Other analyses	17	Report other analyses done—eg analyses of subgroups and interactions, and sensitivity analyses	Supplementary 1, 2
**Discussion**			
Key results	18	Summarise key results with reference to study objectives	23
Limitations	19	Discuss limitations of the study, taking into account sources of potential bias or imprecision. Discuss both direction and magnitude of any potential bias	26-28
Interpretation	20	Give a cautious overall interpretation of results considering objectives, limitations, multiplicity of analyses, results from similar studies, and other relevant evidence	23-26
Generalisability	21	Discuss the generalisability (external validity) of the study results	24-25
**Other information**			
Funding	22	Give the source of funding and the role of the funders for the present study and, if applicable, for the original study on which the present article is based	29-30

*Give information separately for exposed and unexposed groups.

Note: An Explanation and Elaboration article discusses each checklist item and gives methodological background and published examples of transparent reporting. The STROBE checklist is best used in conjunction with this article (freely available on the Web sites of PLoS Medicine at http://www.plosmedicine.org/, Annals of Internal Medicine at http://www.annals.org/, and Epidemiology at http://www.epidem.com/). Information on the STROBE Initiative is available at http://www.strobe-statement.org.

For peer review only

BMJ Open

Association between social isolation and depression onset among older adults: A cross-national longitudinal study in England and Japan

Journal:	*BMJ Open*
Manuscript ID	bmjopen-2020-045834.R2
Article Type:	Original research
Date Submitted by the Author:	19-Feb-2021
Complete List of Authors:	Noguchi, Taiji; National Center for Geriatrics and Gerontology, Department of Social Science, Center for Gerontology and Social Science; Nagoya City University Graduate School of Medical Sciences and Medical School, Department of Public Health<br>Saito, Masashige; Nihon Fukushi University, Faculty of Social Welfare<br>Aida, Jun; Tokyo Medical and Dental University Graduate School of Medical and Dental Sciences, Department of Oral Health Promotion; Tohoku University, Division for Regional Community Development, Liaison Center for Innovative Dentistry<br>Cable, Noriko; University College London, Epidemiology and Public Health<br>Tsuji, Taishi; University of Tsukuba, Faculty of Health and Sport Sciences; Chiba University, Department of Social Preventive Medical Sciences, Center for Preventive Medical Sciences<br>Koyama, Shihoko; Osaka International Cancer Institute, Cancer Control Center<br>Ikeda, Takaaki; Yamagata University Faculty of Medicine Graduate School of Medical Science, Department of Health Policy Science; Tohoku University Graduate School of Dentistry School of Dentistry, Department of International and Community Oral Health<br>Osaka, Ken; Tohoku University Graduate School of Dentistry School of Dentistry, Department of International and Community Oral Health<br>Kondo, Katsunori; Chiba University, Department of Social Preventive Medical Sciences, Center for Preventive Medical Sciences; National Center for Geriatrics and Gerontology, Department of Gerontological Evaluation, Center for Gerontology and Social Science
**Primary Subject Heading**:	Epidemiology
Secondary Subject Heading:	Mental health, Geriatric medicine, Public health
Keywords:	EPIDEMIOLOGY, GERIATRIC MEDICINE, Old age psychiatry < PSYCHIATRY, PUBLIC HEALTH, SOCIAL MEDICINE

For peer review only - http://bmjopen.bmj.com/site/about/guidelines.xhtml

I, the Submitting Author has the right to grant and does grant on behalf of all authors of the Work (as defined in the below author licence), an exclusive licence and/or a non-exclusive licence for contributions from authors who are: i) UK Crown employees; ii) where BMJ has agreed a CC-BY licence shall apply, and/or iii) in accordance with the terms applicable for US Federal Government officers or employees acting as part of their official duties; on a worldwide, perpetual, irrevocable, royalty-free basis to BMJ Publishing Group Ltd ("BMJ") its licensees and where the relevant Journal is co-owned by BMJ to the co-owners of the Journal, to publish the Work in this journal and any other BMJ products and to exploit all rights, as set out in our licence.

The Submitting Author accepts and understands that any supply made under these terms is made by BMJ to the Submitting Author unless you are acting as an employee on behalf of your employer or a postgraduate student of an affiliated institution which is paying any applicable article publishing charge ("APC") for Open Access articles. Where the Submitting Author wishes to make the Work available on an Open Access basis (and intends to pay the relevant APC), the terms of reuse of such Open Access shall be governed by a Creative Commons licence – details of these licences and which Creative Commons licence will apply to this Work are set out in our licence referred to above.

Other than as permitted in any relevant BMJ Author's Self Archiving Policies, I confirm this Work has not been accepted for publication elsewhere, is not being considered for publication elsewhere and does not duplicate material already published. I confirm all authors consent to publication of this Work and authorise the granting of this licence.

For peer review only

For peer review only - http://bmjopen.bmj.com/site/about/guidelines.xhtml

Original research

Title

Association between social isolation and depression onset among older adults: A cross-national longitudinal study in England and Japan

Taiji Noguchi[1,2*], Masashige Saito[3], Jun Aida[4,5], Noriko Cable[6], Taishi Tsuji[7,8], Shihoko Koyama[9], Takaaki Ikeda[10,11], Ken Osaka[11], Katsunori Kondo[1,8]

[1]Center for Gerontology and Social Science, National Center for Geriatrics and Gerontology, Aichi, Japan

[2]Department of Public Health, Nagoya City University Graduate School of Medical Sciences, Aichi, Japan

[3]Faculty of Social Welfare, Nihon Fukushi University, Aichi, Japan

[4]Department of Oral Health Promotion, Graduate School of Medical and Dental Sciences, Tokyo Medical and Dental University, Tokyo, Japan

[5]Division for Regional Community Development, Liaison Center for Innovative Dentistry, Graduate School of Dentistry, Tohoku University, Miyagi, Japan

[6]Department of Epidemiology and Public Health, University College London, London,

For peer review only

UK

[7]Faculty of Health and Sport Sciences, University of Tsukuba, Tokyo, Japan

[8]Department of Social Preventive Medical Sciences, Center for Preventive Medical Sciences, Chiba University, Chiba, Japan

[9]Cancer Control Center, Osaka International Cancer Institute, Osaka, Japan

[10]Department of Health Policy Science, Graduate School of Medical Science, Yamagata University, Yamagata, Japan

[11]Department of International and Community Oral Health, Tohoku University Graduate School of Dentistry, Miyagi, Japan

***Corresponding author**

7-430 Morioka, Obu, Aichi 474-8511, Japan

E-mail: noguchi.taiji0415@gmail.com

Tel: +81-562-46-2311; Fax: +81-562-48-7474

Word count: 3,216 words

For peer review only

ABSTRACT

Objective: Social isolation is a risk factor for depression in older age. However, little is known regarding whether its impact varies depending on country-specific cultural contexts regarding social relationships. The present study examined the association of social isolation with depression onset among older adults in England, which has taken advanced measures against social isolation, and Japan, a super-aged society with a rapidly increasing number of socially isolated people.

Design: Prospective longitudinal study.

Setting: We utilised data from two ongoing studies: the English Longitudinal Study of Ageing (ELSA) and the Japan Gerontological Evaluation Study (JAGES).

Participants: Older adults aged ≥ 65 years without depression at baseline were followed up regarding depression onset for two years (2010/11 to 2012/13) for the ELSA and 2.5 years (2010/11 to 2013) for the JAGES.

Primary outcome measure: Depression was assessed with the Center for Epidemiologic Studies Depression Scale for the ELSA and Geriatric Depression Scale for the JAGES. Multivariable logistic regression analysis was performed to evaluate social isolation using multiple parameters (marital status; interaction with children, relatives, and friends; and social participation).

For peer review only

Results: The data of 3,331 respondents from the ELSA and 33,127 from the JAGES were analysed. Multivariable logistic regression analysis demonstrated that social isolation was significantly associated with depression onset in both countries. In the ELSA, poor interaction with children was marginally associated with depression onset, while in the JAGES, poor interaction with children and no social participation significantly affected depression onset.

Conclusions: Despite variations in cultural background, social isolation was associated with depression onset in both England and Japan. Addressing social isolation to safeguard older adults' mental health must be globally prioritised.

For peer review only

Strengths and limitations of this study

- This is the first cross-national longitudinal study to examine the association between social isolation and depression onset in England, which has taken advanced measures against social isolation, and Japan, a super-aged society with a rapidly increasing number of socially isolated people.
- This study included a large sample of over 3,300 individuals from England and 33,000 individuals from Japan aged 65 years and older.
- A limitation of this study is that we cannot make direct comparisons because of

variations in cohort follow-up periods and depression measurement.

- Another limitation is the use of social support for the evaluation of social contact so as to permit the use of the same social isolation assessment scale in both countries.

For peer review only

INTRODUCTION

With population ageing, there is growing worldwide interest in social issues concerning older adults, including social isolation and the deterioration of physical and mental health. Defined as an objective state in where an individual has few close relationships or limited contact within a community,[1] social isolation is recognised as a social determinant of health with relevance to mortality,[2] cardiovascular diseases,[3] dementia,[4] and mental health.[5, 6] Social isolation is a major risk factor for mental health problems in older age. Several systematic reviews have demonstrated that social isolation is associated with depressive symptoms,[5, 6] which, in turn, are correlated with unhealthy behaviours and reduced access to material resources.[7] Depression, common in later life, is related to adverse health outcomes such as poor quality of life[8] and functional disability.[9] With the high current global burden of depression expected to increase further by 2030,[10] addressing social isolation is an important gerontological issue for protecting mental health among older adults.

The impacts of social isolation on health may vary by country; this could be the result of differences in the social environments related to social networks within and outside the family. A recent study of older adults in England and Japan showed that social isolation is a common risk factor for mortality in both countries, with a greater impact

observed in England; the results are discussed in terms of possible differences between societies that are highly connected and those that are not.[11] In the United Kingdom (UK), in recognition of the impact of social isolation on health and economic loss, the position of 'Minister of Loneliness' was established in 2018, and the country is taking a progressive approach to the elimination of social isolation.[12] In contrast, Japan, now a super-aged society (more than 21% of the population aged 65 or above),[13] is experiencing a rapidly increasing trend in the number of never-married persons and weakening community and neighbourhood relations,[14] leading to a rise in the number of socially isolated individuals.[15] In Japan, the proportion of people who rarely or never spend time with those close to them has been reported to be the highest among Organisation for Economic Co-operation and Development countries. In particular, this figure is much higher than in the UK, which has made advances in tackling social isolation (Japan = 15.3%, UK = 5.0%).[16] Owing to differences in social structures and the contexts surrounding social isolation, the impact of social isolation on depression is expected to vary across countries.

Furthermore, the health effects of social isolation may differ depending on the cultural context of social relationships. In East Asian countries, including Japan, there is a familial norm based on the traditional culture of filial piety,[17] which is often

For peer review only

contrasted with individualism in Western countries.[18, 19] Based on this cultural background, Japanese social support networks may be kinship centred, which may be narrower than the types of social networks prevalent in other countries.[20] However, there is a lack of consensus on the health effects of social relationships based on these cultural differences. A previous cross-national study showed that among English men, friendship-based social relationships had a significant impact on longevity, whereas among Japanese men, this impact was associated with family-based social relationships.[21] In contrast, a study of older adults in the United States (US) and Japan demonstrated that while relationships with children were associated with a low level of depression only in Japan, the presence of spouses was important in both countries, but more so in the US.[22] Another comparative study among adults suggested that social contact with friends benefitted women's mental health in the UK but not in Japan.[23] Thus, the family-oriented nature of East Asian societies does not automatically imply the health importance of family-based relationships, and the roles of individual components of social isolation (family, friends, and others) in the mental health of older adults in each country remain controversial.

As the association between social isolation and depression is often described as bidirectional,[24] longitudinal studies are needed to address temporality. However,

For peer review only

previous cross-national comparative studies have employed only cross-sectional designs.[22, 23] Therefore, using longitudinal data from both countries, the present study aims to investigate the association of social isolation with depression onset in England, which has taken advanced measures against social isolation, and Japan, a super-aged society with a rapidly increasing number of socially isolated people.

METHODS

Sample

This longitudinal study was conducted using data from two ongoing prospective cohort studies: the English Longitudinal Study of Ageing (ELSA) and the Japan Gerontological Evaluation Study (JAGES). The ELSA targets independent-living older adults aged ≥ 50, while JAGES participants are community-dwelling individuals aged ≥ 65 who are ineligible for long-term healthcare insurance benefits.[25] Details of the ELSA and JAGES can be found elsewhere.[26, 27] For the present analysis, we used the two waves of data that most closely corresponded with the timing of our study: wave 5 (2010/2011) to wave 6 (2012/2013) for the ELSA, and wave 1 (2010/2011) to wave 2 (2013) for the JAGES. We harmonised the data by including older adults aged ≥ 65, independent in activities of daily living, and without self-reported dementia. For analysis, respondents

For peer review only

who scored above the cut-off point for depression on each measure in the respective cohort at baseline were excluded and we followed up the onset of depression for two years for the ELSA and 2.5 years for the JAGES.

Depression

Based on a previous cross-national study,[28] depressive symptoms were measured both at baseline and follow-up using eight items from the Center for Epidemiologic Studies Depression Scale (CES-D) in the ELSA[29] and the Geriatric Depression Scale (GDS-15) in the JAGES.[30] To identify possible depressive cases, the CES-D cut-off was ≥ 4 while that for the GDS-15 was ≥ 5.[31, 32] As previously mentioned, respondents with depression at baseline were excluded and we observed the onset of depression during follow-up.

Social isolation

Social isolation levels were assessed using a modified version of the Social Isolation Index (SII).[33–35] The index was computed with respondents given a point if they: (1) were unmarried or living alone, (2) had poor interaction with children (did not live with their children or had no one to provide emotional or instrumental social support), (3) had

For peer review only

poor interaction with relatives (did not have immediate family members providing emotional or instrumental social support), (4) had poor interaction with friends (less than monthly contact or no friends who could provide emotional or instrumental social support), and (5) had no social participation (no participation in any social or religious groups). The total possible score ranged from 0 to 5, with higher scores indicating greater social isolation. The participants were categorised into the following five groups based on their scores: 0, 1, 2, 3, and 4–5 points. We used the total score and the scores of the five sub-components as predictive variables.

Covariates

The covariates included age, gender, educational attainment, household equivalised income, present illness, self-rated health, smoking, and drinking. Age was categorised as 65–69, 70–74, 75–79, 80–84, and ≥ 85. Based on the ages of respondents who had completed formal education, the age of final educational attainment was categorised as ≤ 15 years, 16–18 years, and ≥ 19 years. Household equivalised income was classified into quintiles. Present illness was classified as 'yes' or 'no' for cancer, heart disease, and stroke. Self-rated health was dichotomised as 'poor' and 'good'. Smoking and drinking were dichotomised as 'never/past' and 'current'.

For peer review only

Statistical analysis

We analysed the ELSA and JAGES data separately because of differences in research design, especially sampling approaches. A longitudinal weight was applied to account for survey non-response for the ELSA but not the JAGES as its design does not allow it. First, we calculated descriptive statistics. Second, we conducted a multivariable logistic regression analysis to examine the association between SII score and depression onset and obtained odds ratios (ORs) and 95% confidence intervals (CIs) for depression onset. Model 1 was not adjusted for covariates while Model 2 was adjusted for all covariates. Additionally, we analysed the association between SII sub-components and depression onset, adjusted for all covariates.

To mitigate potential biases resulting from missing information, we used the multiple imputation approach under the missing at random assumption. We generated 20 imputed datasets for the final analysis, which excluded those who met the exclusion criteria and did not respond to the follow-up surveys, using the multiple imputation by chained equations procedure and pooled the results using Rubin's rule.[36]

The significance level was set at $p < 0.05$. We used R (Version 3.5.2 for Windows) for all statistical analyses.

For peer review only - http://bmjopen.bmj.com/site/about/guidelines.xhtml

Ethical considerations

The ELSA investigators received ethical approval for all waves of the study from the National Health Service Research Ethics Committees under the National Research and Ethics Services (MREC/01/2/91). The JAGES protocols were approved by the Ethics Committee on the Research of Human Subjects at Nihon Fukushi University (10-05).

Patient and public involvement

No patients were involved in the development of the research question, study design, or data interpretation.

RESULTS

A total of 3,331 ELSA respondents and 33,127 JAGES respondents were included in the final analysis. Their baseline characteristics are presented in Table 1. The mean age (standard deviation) was 73.6 (6.9) years for the ELSA and 72.4 (5.4) years for the JAGES. Regarding SII scores, the ELSA had the largest number of respondents with 0 and 1 points, while the JAGES had the largest number with 2 and 3 points. In the ELSA, respondents who were older, male, less educated, had a lower income, had heart disease, had poor

For peer review only

184 self-rated health, smoked, consumed little alcohol, and had higher baseline depressive symptom scores and higher SII scores. A similar trend was observed in the JAGES, but here, those who consumed more alcohol had higher SII scores.

For peer review only

Table 1. Respondents' baseline characteristics

	ELSA [a]					JAGES				
	Social Isolation Index score [b]					Social Isolation Index score [b]				
	0	1	2	3	≥ 4	0	1	2	3	≥ 4
	n = 905 (27.2%)	n = 1049 (31.5%)	n = 596 (17.9%)	n = 216 (6.5%)	n = 49 (1.5%)	n = 1402 (4.2%)	n = 5981 (18.0%)	n = 9723 (29.4%)	n = 8735 (26.4%)	n = 2176 (6.6%)
Age (years), (%)										
65–69	41.0	36.4	29.9	27.6	28.3	38.6	40.5	38.0	37.0	35.8
70–74	26.1	28.3	28.8	29.3	23.6	32.1	32.5	31.6	31.0	30.4
75–79	20.9	17.5	19.3	15.8	20.4	19.2	18.3	19.5	20.3	21.9
80–84	9.1	12.2	12.5	14.5	15.5	8.3	6.7	8.3	9.0	8.8
≥ 85	2.8	5.7	9.5	12.8	12.2	1.9	2.0	2.6	2.7	3.1
Gender, (%)										
Men	50.0	46.1	45.3	51.9	65.8	27.2	35.1	47.0	64.4	66.1
Women	50.0	53.9	54.7	48.1	34.2	72.8	64.9	53.0	35.6	33.9
Educational attainment (years), (%)										
≤ 15	44.1	51.3	52.3	60.9	69.6	42.9	38.1	37.9	38.7	48.6
16–18	35.0	33.8	34.6	27.5	14.1	40.9	40.8	39.3	36.8	31.1
≥ 19	17.8	12.5	11.0	10.3	14.0	15.7	20.4	21.8	23.3	18.5
Missing	3.2	2.4	2.1	1.3	2.3	0.5	0.7	0.9	1.2	1.8
Household equivalised income, (%)										
1st quintile (lowest)	11.4	18.3	23.2	31.2	14.4	7.4	10.9	13.9	15.6	21.3
2nd quintile	21.5	23.7	26.1	24.1	29.6	13.2	13.5	15.0	15.5	16.4
3rd quintile	20.9	21.8	18.9	19.5	27.1	22.5	30.6	30.3	30.6	28.0
4th quintile	21.9	19.9	18.1	16.5	16.6	14.3	13.6	12.7	11.3	9.5
5th quintile (highest)	22.7	15.0	12.7	8.3	12.4	31.5	22.5	19.2	16.8	12.9
Missing	1.5	1.3	0.9	0.4	0.0	11.1	8.9	8.9	10.2	11.9
Cancer, (%)										
No	96.5	95.8	97.1	97.5	96.5	91.0	91.2	90.8	90.1	89.7
Yes	3.4	4.2	2.9	2.5	3.5	3.2	3.1	3.1	3.4	3.7
Missing	0.1	0.0	0.0	0.0	0.0	5.8	5.7	6.1	6.5	6.6
Heart disease, (%)										
No	90.7	86.9	88.4	88.3	77.3	91.0	91.2	90.8	90.1	89.7
Yes	9.3	13.0	11.6	11.7	22.7	3.2	3.1	3.1	3.4	3.7
Missing	0.0	0.1	0.0	0.0	0.0	5.8	5.7	6.1	6.5	6.6

For peer review only

Stroke, (%)										
No	96.3	96.1	97	94.4	96.6	93.3	93.6	93.1	92.5	92.2
Yes	3.7	3.8	3.0	5.6	3.4	0.9	0.8	0.8	1.0	1.2
Missing	0	0.1	0.0	0.0	0.0	5.8	5.7	6.1	6.5	6.6
Self-rated health, (%)										
Good	86.4	79.9	78.8	75.4	71.3	92.0	91.7	90.6	90.2	87.2
Poor	13.6	20.1	21.2	24.6	28.7	7.2	7.7	8.6	9.0	12.2
Missing	0.0	0.0	0.0	0.0	0.0	0.8	0.5	0.8	0.8	0.6
Smoking, (%)										
Never/past	95.5	93.1	89.8	82.9	89.4	86.4	86.1	82.9	81.5	78.4
Current	4.5	6.9	10.2	17.1	10.6	6.4	7.4	9.5	11.3	14.2
Missing	0.0	0.0	0.0	0.0	0.0	7.1	6.5	7.5	7.2	7.3
Drinking, (%)										
Never/past	7.5	11.8	14.9	25.8	17.3	65.0	60.0	56.5	50.4	55.0
Current	91.3	87.0	82.9	72.6	73.6	30.2	35.7	38.6	44.7	40.4
Missing	1.3	1.2	2.2	1.6	9.2	4.8	4.3	4.9	4.9	4.6
CES-D score at baseline, (%)										
0	57.2	52.0	45.5	42.2	54.0					
1	27.3	26.2	28.6	31.8	25.5					
2	9.8	13.8	17.1	11.9	15.1					
3	5.7	8.0	8.8	14.2	5.4					
GDS score at baseline, (%)										
0						20.5	30.4	29.0	26.4	24.3
1						25.5	28.4	28.6	26.9	27.0
2						22.5	20.0	19.8	21.5	21.1
3						17.5	13.1	13.5	14.7	15.9
4						14.1	8.1	9.1	10.5	11.7

ELSA, English Longitudinal Study of Ageing; JAGES, Japan Gerontological Evaluation Study; CES-D, Center for Epidemiologic Studies Depression Scale; GDS, Geriatric Depression Scale

[a] ELSA data after sampling weight

[b] Missing data: ELSA, n = 516; JAGES, n = 5,110

For peer review only

Table 2 presents the description of social isolation and depression onset. At follow-up, 201 (6.0%) ELSA respondents and 4,456 (13.5%) JAGES respondents exhibited depression onset. In both studies, higher SII scores were associated with an increased risk of depression onset. Regarding SII sub-components, ELSA respondents who were unmarried or living alone were more likely to have depression, while this was the case with JAGES respondents with no social participation.

For peer review only

199 **Table 2. Description of social isolation status and depression onset**

		ELSA [a]		JAGES	
		CES-D score at follow-up		GDS score at follow-up	
		< 4	≥ 4	< 5	≥ 5
		n = 3130 (94.0%)	n = 201 (6.0%)	n = 28671 (86.5%)	n = 4456 (13.5%)
Social Isolation Index score, (%)					
0		27.5	13.5	4.4	3.0
1		31.2	29.3	18.6	14.3
2		17.9	18.9	29.8	26.3
3		6.5	10.1	26.2	27.5
≥ 4		1.5	2.9	6.1	9.3
Missing		15.4	25.3	14.8	19.7
Social Isolation Index sub-components, (%)					
Unmarried or living alone	No	71.9	58.5	88.3	85.2
	Yes	28.1	41.5	10.2	12.7
	Missing	0.0	0.0	1.4	2.1
Poor interaction with children	No	81.8	73.4	26.6	25.0
	Yes	12.5	14.7	71.7	72.9
	Missing	5.7	11.8	1.7	2.1
Poor interaction with relatives	No	76.3	68.1	41.1	38.7
	Yes	17.3	18.6	54.4	56.0
	Missing	6.4	13.4	4.5	5.4
Poor interaction with friends	No	72.2	66.0	37.1	31.2
	Yes	18.4	21.6	58.2	63.8
	Missing	9.4	12.4	4.6	5.0
No social participation	No	61.7	46.3	75.0	63.9
	Yes	28.4	32.5	13.0	20.0
	Missing	10.0	21.2	11.7	16.1

200 ELSA, English Longitudinal Study of Ageing; JAGES, Japan Gerontological Evaluation
201 Study; CES-D, Center for Epidemiologic Studies Depression Scale; GDS, Geriatric
202 Depression Scale

203 [a] ELSA data after sampling weight

For peer review only

204 Table 3 depicts the association between SII scores and depression onset.
205 Multivariable analysis showed that higher SII scores were associated with a higher risk
206 of depression onset in both studies after adjusting for all covariates. In the ELSA, the OR
207 of depression onset was significantly higher from a score ≥ one point (OR [95% CI]
208 compared with zero points, one: 1.68 [1.02–2.75], two: 1.77 [1.03–3.05], three: 2.64
209 [1.37–5.12], ≥ four: 4.01 [1.43–11.22], *p* for trend = 0.015). In the JAGES, as SII scores
210 increased, the OR of depression onset gradually increased, reaching significance at ≥
211 three points (OR [95% CI] compared with zero points, one: 1.10 [0.89–1.35], two: 1.15
212 [0.94–1.40], three: 1.28 [1.04–1.56], ≥ four: 1.48 [1.18–1.85], *p* for trend < 0.001). These
213 results showed almost the same tendency as the complete case analysis without multiple
214 imputation (Supplementary Table 1).

For peer review only

Table 3. Association between social isolation and depression onset: multivariable logistic regression analysis

	ELSA		JAGES	
	Crude OR (95% CI)	Adjusted OR (95% CI) [a]	Crude OR (95% CI)	Adjusted OR (95% CI) [a]
Social Isolation Index score				
0	1.00 (reference)	1.00 (reference)	1.00 (reference)	1.00 (reference)
1	1.92** (1.19–3.10)	1.68* (1.02–2.75)	1.14 (0.94–1.39)	1.10 (0.89–1.35)
2	2.15** (1.28–3.62)	1.77* (1.03–3.05)	1.32** (1.09–1.60)	1.15 (0.94–1.40)
3	3.19*** (1.73–5.90)	2.64** (1.37–5.12)	1.57*** (1.30–1.90)	1.28* (1.04–1.56)
≥ 4	3.85** (1.46–10.18)	4.01** (1.43–11.22)	2.26*** (1.83–2.79)	1.48*** (1.18–1.85)
	p for trend < 0.001	*p* for trend = 0.015	*p* for trend < 0.001	*p* for trend < 0.001

* $p < 0.05$; ** $p < 0.01$; *** $p < 0.001$

ELSA, English Longitudinal Study of Ageing; JAGES, Japan Gerontological Evaluation Study; CI, confidence interval; OR, odds ratio

[a] Adjusted for age, gender, educational attainment, household equivalised income, present illness (cancer, heart disease, and stroke), self-rated health, smoking, drinking, and depression score at baseline (Center for Epidemiologic Studies Depression Scale for the ELSA and Geriatric Depression Scale for the JAGES).

For peer review only

221 Table 4 presents the associations of SII sub-components with depression onset.
222 In the ELSA, sub-components were not significant, although poor interaction with
223 children was marginally significant (OR [95% CI], with 'none' as the reference;
224 unmarried or living alone: 1.13 [0.80–1.60], poor interaction with children: 1.55 [1.00–
225 2.41], poor interaction with relatives: 1.24 [0.79–1.94], poor interaction with friends: 1.15
226 [0.77–1.71], no social participation: 1.22 [0.80–1.87]). In the JAGES, poor interaction
227 with children and no social participation were significantly associated with depression
228 onset after adjusting for all covariates (OR [95% CI], with 'none' as the reference;
229 unmarried or living alone: 1.11 [1.00–1.24], poor interaction with children: 1.09 [1.01–
230 1.19], poor interaction with relatives: 1.04 [0.96–1.12], poor interaction with friends: 1.03
231 [0.95–1.11], no social participation: 1.28 [1.17–1.40]). These results were similar to those
232 obtained from the complete case analysis (Supplementary Table 2).

For peer review only

Table 4. Association between sub-components of social isolation and depression onset: multivariable logistic regression analysis

	ELSA	JAGES
	Adjusted OR (95% CI) [a]	Adjusted OR (95% CI) [a]
Social Isolation Index sub-components (reference: none)		
Unmarried or living alone	1.13 (0.80–1.60)	$1.11^{\dagger}$ (1.00–1.24)
Poor interaction with children	$1.55^{\dagger}$ (1.00–2.41)	1.09^{*} (1.01–1.19)
Poor interaction with relatives	1.24 (0.79–1.94)	1.04 (0.96–1.12)
Poor interaction with friends	1.15 (0.77–1.71)	1.03 (0.95–1.11)
No social participation	1.22 (0.80–1.87)	1.28^{***} (1.17–1.40)

$^{*}\ p < 0.05$; $^{**}\ p < 0.01$; $^{***}\ p < 0.001$; $^{\dagger}\ p < 0.1$

ELSA, English Longitudinal Study of Ageing; JAGES, Japan Gerontological Evaluation Study; CI, confidence interval; OR, odds ratio

[a] Adjusted for age, gender, educational attainment, equivalent income, present illness (cancer, heart disease, and stroke), self-rated health, smoking, drinking, and depression score at baseline (Center for Epidemiologic Studies Depression Scale for the ELSA and Geriatric Depression Scale for the JAGES).

For peer review only

For peer review only - http://bmjopen.bmj.com/site/about/guidelines.xhtml

DISCUSSION

To the best of our knowledge, this is the first cross-national longitudinal study of the association of social isolation with depression among older English and Japanese adults. Social isolation was significantly associated with depression onset in both countries. Our results support previous longitudinal findings on social relationships and mental health among older adults in England[37] and Japan.[38] Using data frames that were similar with regard to assessment and covariates, we demonstrated that social isolation is a common risk factor for depression in England and Japan, despite country-specific cultural differences regarding social relationships. Thus, our results suggest that to safeguard the mental health of older adults, addressing social isolation is a global need.

The association between social isolation and depression was somewhat stronger in England than in Japan. These results are similar to a previous report concerning mortality among older adults in England and Japan.[11] Although we cannot make direct comparisons due to variations in cohort follow-up periods and depression measurement, there are several possible reasons for this pattern of findings. The impact of social factors could differ depending on the group and society to which one belongs. This is best understood in the context of the concept of relative deprivation.[39] In other words, higher levels of relative social isolation may induce greater psychological stress. A previous

For peer review only

study showed that rich community ties and cohesion were protective factors for health but could have a negative effect on those who were not socially involved.[40] Being isolated in a connected society such as the UK may represent a more severe condition, with a stronger negative impact on mental health.

Our results revealed that poor interaction with children was significant with regard to depression onset in Japan. In England, while the association was marginal, of the components of social isolation, poor interaction with children had the greatest effect. The lack of interaction with children could have an adverse effect on the mental health of older adults in both countries. Previous studies in England[41] and Japan[22] have reported that social support from children can contribute to alleviating depression, and our results point in the same direction. Older adults without children can be considered a vulnerable group, because adult children, in particular, are often the main source of positive social support for older parents.[42] Older parents have certain expectations with regard to receiving support from their children, and situations wherein these expectations are not met may lead to depressive mood.[43] However, a previous study reported no association between the presence of children and depression among older adults in the US.[22] Owing to strong spousal relationships in the US, the effect of the presence of children might be relatively small. Thus, our study confirmed the adverse effects of poor

For peer review only

interaction with children common to England and Japan, but international generalizability can only be established based on further research considering the cultural background of family relationships in individual countries.

Although traditionally Japan is a country in which adult children are expected to demonstrate reciprocity with their parents based on the strong family and kinship-based cultural background,[44] in this study, the effect of interaction with children on depression was relatively modest. In recent years, with trends such as adult children commonly living apart from their parents after marriage[45] and the development of public long-term care services for the ageing population,[46] Japan's family system has become less traditional. Therefore, interaction with children may not be as essential to the health of older adults as before. However, despite these cultural transitions, we believe that interaction with children has some value with regard to preventing depression in old age in Japan.

Social participation was a strong protective factor for depression onset in Japan, whereas there was no association in England, although the OR was somewhat greater. Several previous studies have reported that social participation helps prevent depression onset.[37, 47–49] Our results pertaining to Japan support these reports. However, the protective effects of social participation on mental health vary depending on the type of

For peer review only

organisation with which an individual is involved,[48] the individual's attitude towards participation,[48] and the duration[37] and frequency[49] of participation. Regarding the present study, in the English context, the role of social participation in depression prevention might have been unidentifiable due to differences in the effects of these participation contexts. We took into account only social participation, without delving into specific types. Thus, the context of effective social participation, such as type, duration, and role in the organisation in both countries, requires further investigation. Despite these challenges, our findings suggest that in Japan, social isolation prevention measures based on the promotion of social participation could be beneficial for safeguarding the mental health of older adults.

This study has several strengths. First, it is the first cross-national population-level investigation of the association of social isolation with depression onset using a unified data frame. Second, by using two longitudinal datasets, we were able to examine the prospective association between social isolation and depression. Third, the use of large-scale data allowed us to detect the effects of relatively rare situations of severe social isolation.

However, certain limitations cannot be ignored. First, the measurement of depression in the two cohorts was not the same. Therefore, we could not directly compare

For peer review only

depression onset in the two countries. However, these measurements were also used in a previous cross-national comparison study in England and Japan,[28] and we were able to examine the association between social isolation and depression onset in both countries using the same data frame. Second, we used social support for the assessment of social contact for some items in order to be able to use the same SII. Therefore, cultural differences in expectations regarding the receipt of social support in both countries might have caused information biases. For instance, expectations regarding social support from relatives could originally have been higher in Japan,[44] leading to overestimation of social isolation levels. Third, regarding the items of the SII, the questions and their response options in the ELSA and JAGES were not exactly the same, nor were they strictly authorised through procedures such as reverse translation and confirming reliability and validity. However, we believe it is certainly meaningful to evaluate social isolation using the same framework. Finally, there were differences in study design in the data from the two cohorts, such as sampling method and follow-up period. We, therefore, made efforts to harmonise the data: those aged ≤ 64, with dementia, and dependent in activities of daily living were excluded from the analysis. Moreover, the ELSA presents nationally representative population data, while the JAGES does not. However, the JAGES sample is representative of areas from a nationwide ageing study in which

For peer review only

approximately one-fifth of all prefectures (nine out of 47) were enrolled. Even so, unlike the ELSA, analysis in the JAGES does not use sampling weights, which may lead to selection bias.

CONCLUSION

We examined the association between social isolation and depression onset among older adults in England and Japan, who experience different cultural contexts regarding social relationships, and found a significant association in both countries; we also observed that in England, poor interaction with children was marginally associated, and in Japan, poor interaction and lack of social participation were significantly associated with depression. Tackling social isolation must be prioritised to safeguard the mental health of older adults worldwide. Particularly in Japan, the promotion of interaction with children and social participation could be key factors in addressing social isolation.

ACKNOWLEDGEMENTS

We wish to express our deepest gratitude to the members in the Japan Gerontological Evaluation Study. We would also like to thank everyone who participated in the surveys.

For peer review only

AUTHORS' CONTRIBUTIONS

All authors contributed to the conception and design of this study. Data collection was primarily conducted by MS, JA, NC, KO, and KK. Analyses were performed by TN, MS, JA, TT, SK, and TI. TN prepared the initial manuscript and MS, JA, NC, TT, SK, TI, KO, and KK significantly contributed to revising it. All authors read and approved the final manuscript.

FUNDING STATEMENT

This work was supported by a grant from the Grants-in-Aid for Scientific Research (18KK0057, 19K24277) from the Japan Society for the Promotion of Science. The English Longitudinal Study of Ageing was developed by a team of researchers based at the University College London, NatCen Social Research, and the Institute for Fiscal Studies. The data were collected by NatCen Social Research. The funding is currently provided by the National Institute of Aging (R01AG017644) and a consortium of UK government departments coordinated by the National Institute for Health Research. The Japan Gerontological Evaluation Study is supported by the Ministry of Education, Culture, Sports, Science and Technology-Japan-Supported Program for the Strategic Research Foundation at Private Universities (2009-2013), Japan Society for the Promotion of

For peer review only

Science KAKENHI Grant Numbers (JP18390200, JP22330172, JP22390400, JP23243070, JP23590786, JP23790710, JP24390469, JP24530698, JP24683018, JP25253052, JP25870573, JP25870881, JP26285138, JP26882010, JP15H01972), Health Labour Sciences Research Grants (H22-Choju-Shitei-008, H24-Junkanki -Ippan-007, H24-Chikyukibo-Ippan-009, H24-Choju-Wakate-009, H25-Kenki-Wakate-015, H25-Choju-Ippan-003, H26-Irryo-Shitei-003 [Fukkou], H26-Choju-Ippan-006, H27-Ninchisyou-Ippan-001, H28-chojuIppan-002, H28-Ninchisho-Ippan-002, H30-Kenki-Ippan-006, H30-Junkankitou-Ippan-004), Japan Agency for Medical Research and Development (JP17dk0110017, JP18dk0110027, JP18ls0110002, JP18le0110009, JP19dk0110034), Research Funding for Longevity Sciences from the National Center for Geriatrics and Gerontology (24-17, 24-23, 29-42, 30-22, 20-40), Open Innovation Platform with Enterprises, Research Institute and Academia (OPERA, JPMJOP1831) from the Japan Science and Technology Agency, and a Research Grant from the Health Science Center Foundation (2019-2020). The funding sources had no role in the study design, data collection and analysis, decision to publish or preparation of the manuscript.

DATA SHARING STATEMENT

For the JAGES, all enquiries are to be addressed to the data management committee via

For peer review only

e-mail: dataadmin.ml@jages.net. All JAGES datasets have ethical or legal restrictions for public deposition because of the inclusion of sensitive information about the human participants. Regarding the ELSA, data are available in an open-access repository at http://www. dataarchive.ac.uk/.

COMPETING INTERESTS STATEMENT

The authors declare no conflict of interest.

REFERENCES

1. Townsend P. The family life of old people: an investigation in East London. *Sociol Rev* 1955;3:175–95. doi: 10.1111/j.1467-954X.1955.tb01052.x

2. Holt-Lunstad J, Smith TB, Layton JB. Social relationships and mortality risk: a meta-analytic review. *PLoS Med* 2010;7:e1000316. doi:10.1371/journal.pmed.1000316

3. Valtorta NK, Kanaan M, Gilbody S, et al. Loneliness and social isolation as risk factors for coronary heart disease and stroke: systematic review and meta-analysis of longitudinal observational studies. *Heart* 2016;102:1009–16. doi:10.1136/heartjnl-2015-308790

4. Livingston G, Sommerlad A, Orgeta V, et al. Dementia prevention, intervention, and

care. *Lancet* 2017;390:2673–734. doi:10.1016/s0140-6736(17)31363-6

5. Leigh-Hunt N, Bagguley D, Bash K, et al. An overview of systematic reviews on the public health consequences of social isolation and loneliness. *Public Health* 2017;152:157–71. doi:10.1016/j.puhe.2017.07.035

6. Santini ZI, Koyanagi A, Tyrovolas S, et al. The association between social relationships and depression: a systematic review. *J Affect Disord* 2015;175:53–65. doi:10.1016/j.jad.2014.12.049

7. Kawachi I, Berkman LF. Social ties and mental health. *J Urban Health* 2001;78:458–67. doi:10.1093/jurban/78.3.458

8. Sivertsen H, Bjorklof GH, Engedal K, et al. Depression and quality of life in older persons: a review. *Dement Geriatr Cogn Disord* 2015;40:311–39. doi:10.1159/000437299

9. Chen CM, Mullan J, Su YY, et al. The longitudinal relationship between depressive symptoms and disability for older adults: a population-based study. *J Gerontol A Biol Sci Med Sci* 2012;67:1059–67. doi:10.1093/gerona/gls074

10. World Federation for Mental Health. Depression: a global crisis. World Mental Health Day, October 10 2012. 2012. https://www.who.int/mental_health/management/depression/wfmh_paper_depre

For peer review only

ssion_wmhd_2012.pdf (accessed 7 Feb 2020).

11. Saito M, Aida J, Cable N, et al. Cross-national comparison of social isolation and mortality among older adults: a 10-year follow-up study in Japan and England. *Geriatr Gerontol Int* 2020 doi: 10.1111/ggi.14118

12. HM Government. A connected society: a strategy for tackling loneliness – laying the foundations for change. 2018. https://assets.publishing.service.gov.uk/government/uploads/system/uploads/attachment_data/file/750909/6.4882_DCMS_Loneliness_Strategy_web_Update.pdf (accessed 12 Oct 2020).

13. Muramatsu N, Akiyama H. Japan: super-aging society preparing for the future. *Gerontologist* 2011;51:425–32. doi: 10.1093/geront/gnr067

14. National Institute of Population and Social Security Research. Household projections for Japan. http://www.ipss.go.jp/pp-ajsetai/e/hprj_98/hprj_98.html (accessed 12 Oct 2020).

15. Murayama H, Shibui Y, Fukuda Y, et al. A new crisis in Japan-social isolation in old age. *J Am Geriatr Soc* 2011;59:2160–2. doi:10.1111/j.1532-5415.2011.03640.x

16. Organisation for Economic Co-operation and Development. Society at a Glance: OECD Social Indicators. Paris: OECD Publishing 2005.

For peer review only

17. Takagi E, Silverstein M. Intergenerational coresidence of the Japanese elderly:are cultural norms proactive or reactive? *Research on Aging* 2006;28:473–92. doi: 10.1177/0164027506287788

18. Knight B, Robinson Shurgot G, Longmire C, et al. Cross cultural issues in caregiving for persons with dementia: do familism values reduce burden and distress? *Aging International* 2002;27:70–94. doi: 10.1007/s12126-003-1003-y

19. Knight BG, Sayegh P. Cultural values and caregiving: the updated sociocultural stress and coping model. *J Gerontol B Psychol Sci Soc Sci* 2009;65B(1):5–13. doi: 10.1093/geronb/gbp096 %

20. Cabinet Office. International Comparative Survey on the Life and Awareness of the Elderly in 2015. Cabinet Office, Tokyo. 2015. https://www8.cao.go.jp/kourei/ishiki/h27/zentai/index.html (accessed 30 Jan 2021).

21. Aida J, Cable N, Zaninotto P, et al. Social and behavioural determinants of the difference in survival among older adults in Japan and England. *Gerontology* 2018;64:266–77. doi:10.1159/000485797

22. Sugisawa H, Shibata H, Hougham GW, et al. The impact of social ties on depressive symptoms in U.S. and Japanese elderly. *J Soc Issues* 2003;58:785–804.

For peer review only

doi:10.1111/1540-4560.00290

23. Cable N, Chandola T, Lallukka T, et al. Country specific associations between social contact and mental health: evidence from civil servant studies across Great Britain, Japan and Finland. *Public Health* 2016;137:139–46. doi: 10.1016/j.puhe.2015.10.013

24. Santini ZI, Jose PE, York Cornwell E, et al. Social disconnectedness, perceived isolation, and symptoms of depression and anxiety among older Americans (NSHAP): a longitudinal mediation analysis. *Lancet Public Health* 2020;5:e62–70. doi:10.1016/s2468-2667(19)30230-0

25. Mossialos E, Djordjevic A, Osborn R, et al. International profiles of health care systems. 2017. https://www.commonwealthfund.org/sites/default/files/documents/___media_files_publications_fund_report_2017_may_mossialos_intl_profiles_v5.pdf (accessed 12 Oct 2020).

26. Kondo K. Progress in aging epidemiology in Japan: the JAGES Project. *J Epidemiol* 2016;26:331–6. doi:10.2188/jea.JE20160093

27. Steptoe A, Breeze E, Banks J, et al. Cohort profile: the English longitudinal study of ageing. *Int J Epidemiol* 2013;42:1640–8. doi: 10.1093/ije/dys168

For peer review only

28. Cable N, Chandola T, Aida J, et al. Can sleep disturbance influence changes in mental health status? Longitudinal research evidence from ageing studies in England and Japan. *Sleep Med* 2017;30:216–21. doi:10.1016/j.sleep.2016.11.017

29. Marmot M, Banks J, Blundell R, et al. Health, Wealth and Lifestyles of the Older Population in England: The 2002 English Longitudinal Study of Ageing. London: Institute for Fiscal Studies 2003.

30. Wada T, Ishine M, Kita T, et al. Depression screening of elderly community-dwelling Japanese. *J Am Geriatr Soc* 2003;51:1328–9. doi: 10.1046/j.1532-5415.2003.514207.x

31. Gallagher D, Kiss A, Lanctot K, et al. Depressive symptoms and cognitive decline: a longitudinal analysis of potentially modifiable risk factors in community dwelling older adults. *J Affect Disord* 2016;190:235–40. doi:10.1016/j.jad.2015.09.046

32. Burke WJ, Roccaforte WH, Wengel SP. The short form of the Geriatric Depression Scale: a comparison with the 30-item form. *J Geriatr Psychiatry Neurol* 1991;4:173–8. doi:10.1177/089198879100400310

33. Ikeda T, Cable N, Saito M, et al. Association between social isolation and smoking in Japan and England. *J Epidemiol* 2020 doi: 10.2188/jea.JE20200138

34. Tsuji T, Saito M, Ikeda T, et al. Change in the prevalence of social isolation among

For peer review only

the older population from 2010 to 2016: a repeated cross-sectional comparative study of Japan and England. *Arch Gerontol Geriatr* 2020;91:104237. doi: 10.1016/j.archger.2020.104237

35. Shankar A, McMunn A, Banks J, et al. Loneliness, social isolation, and behavioral and biological health indicators in older adults. *Health Psychol* 2011;30:377–85. doi:10.1037/a0022826

36. White IR, Royston P, Wood AM. Multiple imputation using chained equations: issues and guidance for practice. *Stat Med* 2011;30:377–99. doi:10.1002/sim.4067

37. Cruwys T, Dingle GA, Haslam C, et al. Social group memberships protect against future depression, alleviate depression symptoms and prevent depression relapse. *Soc Sci Med* 2013;98:179–86. doi:10.1016/j.socscimed.2013.09.013

38. Koizumi Y, Awata S, Kuriyama S, et al. Association between social support and depression status in the elderly: results of a 1-year community-based prospective cohort study in Japan. *Psychiatry Clin Neurosci* 2005;59:563–9. doi:10.1111/j.1440-1819.2005.01415.x

39. Smith HJ, Pettigrew TF, Pippin GM, et al. Relative deprivation: a theoretical and meta-analytic review. *Pers Soc Psychol Rev* 2012;16:203–32. doi:10.1177/1088868311430825

For peer review only

40. Amemiya A, Saito J, Saito M, et al. Social capital and the improvement in functional ability among older people in Japan: a multilevel survival analysis using JAGES data. *Int J Environ Res Public Health* 2019;16:1310. doi:10.3390/ijerph16081310

41. Stafford M, McMunn A, Zaninotto P, et al. Positive and negative exchanges in social relationships as predictors of depression: evidence from the English Longitudinal Study of Aging. *J Aging Health* 2011;23:607–28. doi:10.1177/0898264310392992

42. Koropeckyj-Cox T. Loneliness and depression in middle and old age: are the childless more vulnerable? *J Gerontol B Psychol Sci Soc Sci* 1998;53:S303–12. doi: 10.1093/geronb/53b.6.s303

43. Lee GR, Netzer JK, Coward RT. Depression among older parents: the role of intergenerational exchange. *J Marriage Family* 1995;57:823–33. doi: 10.2307/353935

44. Brown JW, Liang J, Krause N, et al. Transitions in living arrangements among elders in Japan: does health make a difference? *J Gerontol B Psychol Sci Soc Sci* 2002;57:S209–20. doi: 10.1093/geronb/57.4.s209

45. Takagi E, Silverstein M. Purchasing piety? Coresidence of married children with their older parents in Japan. *Demography* 2011;48:1559–79. doi:10.1007/s13524-011-

For peer review only

0053-0

46. Tsutsui T, Muramatsu N. Care-needs certification in the long-term care insurance system of Japan. *J Am Geriatr Soc* 2005;53(3):522–7. doi: 10.1111/j.1532-5415.2005.53175.x

47. Takagi D, Kondo K, Kawachi I. Social participation and mental health: moderating effects of gender, social role and rurality. *BMC Public Health* 2013;13:701. doi:10.1186/1471-2458-13-701

48. Tomioka K, Kurumatani N, Hosoi H. Positive and negative influences of social participation on physical and mental health among community-dwelling elderly aged 65-70 years: a cross-sectional study in Japan. *BMC Geriatr* 2017;17:111. doi: 10.1186/s12877-017-0502-8

49. Choi E, Han KM, Chang J, et al. Social participation and depressive symptoms in community-dwelling older adults: emotional social support as a mediator. *J Psychiatr Res* 2020. doi: 10.1016/j.jpsychires.2020.10.043

For peer review only

Supplementary Table 1. Association between social isolation and depression onset, by complete case analysis

		ELSA		JAGES	
		Crude OR (95% CI)	Adjusted OR (95% CI)	Crude OR (95% CI)	Adjusted OR (95% CI)
Social Isolation Index score	0	1.00 (reference)	1.00 (reference)	1.00 (reference)	1.00 (reference)
	1	1.92** (1.19–3.10)	1.56† (0.94–2.60)	1.14 (0.94–1.39)	1.11 (0.87–1.41)
	2	2.15** (1.28–3.62)	1.71† (0.98–2.99)	1.32** (1.09–1.60)	1.20 (0.95–1.51)
	3	3.19*** (1.73–5.90)	2.50** (1.27–4.90)	1.57*** (1.30–1.90)	1.30* (1.03–1.64)
	≥ 4	3.85** (1.46–10.18)	5.17** (1.83–14.66)	2.26*** (1.83–2.79)	1.47** (1.13–1.91)
		p for trend < 0.001	p for trend < 0.001	p for trend < 0.001	p for trend < 0.001
Age (years)	65–69		1.00 (reference)		1.00 (reference)
	70–74		1.37 (0.83–2.25)		1.16** (1.05–1.30)
	75–79		1.24 (0.71–2.14)		1.31*** (1.16–1.48)
	80–84		1.66† (0.92–2.99)		1.60*** (1.37–1.88)
	≥ 85		0.97 (0.45–2.09)		1.79*** (1.39–2.30)
Gender	Male		1.00 (reference)		1.00 (reference)
	Female		1.58* (1.07–2.34)		1.00 (0.90–1.11)
Educational attainment (years)	< 15		1.00 (reference)		1.00 (reference)
	16–18		1.05 (0.68–1.60)		0.81*** (0.74–0.89)
	≥ 19		1.30 (0.70–2.41)		0.68*** (0.60–0.78)
Household equivalised income	1st quintile (lowest)		1.00 (reference)		1.00 (reference)
	2nd quintile		0.70 (0.40–0.21)		0.95 (0.83–1.09)
	3rd quintile		1.11 (0.66–1.87)		0.76*** (0.67–0.87)
	4th quintile		0.82 (0.45–1.50)		0.67*** (0.57–0.79)
	5th quintile (highest)		0.84 (0.42–1.68)		0.65*** (0.56–0.76)

For peer review only

Cancer	No	1.00 (reference)	1.00 (reference)
	Yes	0.95 (0.32–2.81)	1.16 (0.93–1.43)
Heart disease	No	1.00 (reference)	1.00 (reference)
	Yes	1.13 (0.67–1.92)	1.20 (1.05–1.37)
Stroke	No	1.00 (reference)	1.00 (reference)
	Yes	1.3 (0.56–3.02)	1.18 (0.80–1.73)
Self-rated health	Good	1.00 (reference)	1.00 (reference)
	Poor	1.71* (1.13–2.59)	1.63*** (1.44–1.85)
CES-D score at baseline		1.90*** (1.60–2.25)	
GDS score at baseline			1.92*** (1.86–1.99)
Smoking	Never/past	1.00 (reference)	1.00 (reference)
	Current	0.76 (0.76–1.56)	1.29*** (1.12–1.48)
Drinking	Never/past	1.00 (reference)	1.00 (reference)
	Current	1.41 (0.81 – 2.44)	1.02 (0.93 – 1.13)

*, $p < 0.05$; **, $p < 0.01$; ***, $p < 0.001$; †, $p < 0.1$

ELSA, English Longitudinal Study of Ageing; JAGES, Japan Gerontological Evaluation Study; CES-D, Center for Epidemiologic Studies Depression Scale; CI, confidence interval; GDS, Geriatric Depression Scale; OR, odds ratio

For peer review only

Supplementary Table 2. Association between sub-components of social isolation and depression onset, by complete case analysis

		ELSA		JAGES	
		Crude OR (95% CI)	Adjusted OR (95% CI)	Crude OR (95% CI)	Adjusted OR (95% CI)
Social Isolation Index sub-components (ref; none)					
Unmarried or living alone		1.91*** (1.34–2.72)	1.27 (0.84–1.93)	1.33*** (1.18–1.49)	1.02 (0.88–1.19)
Poor interaction with children		1.08 (0.68–1.74)	1.51 (0.91–2.50)	1.01 (0.98–1.10)	1.13* (1.02–1.25)
Poor interaction with relatives		1.25 (0.83–1.90)	1.33 (0.85–2.08)	1.05 (0.97–1.13)	0.98 (0.90–1.08)
Poor interaction with friends		1.19 (0.78–1.80)	1.21 (0.78–1.87)	1.23 (1.14–1.33)	1.11* (1.01–1.22)
No social participation		1.54* (1.08–2.19)	1.46† (0.98–2.16)	1.70 (1.56–1.86)	1.22*** (1.09–1.36)
Age (years)	65–69		1.00 (reference)		1.00 (reference)
	70–74		1.37 (0.83–2.25)		1.17** (1.05–1.30)
	75–79		1.25 (0.72–2.17)		1.31*** (1.16–1.49)
	80–84		1.68† (0.92–3.04)		1.60*** (1.36–1.88)
	≥ 85		1.00 (0.46–2.21)		1.78*** (1.38–2.30)
Gender	Male		1.00 (reference)		1.00 (reference)
	Female		1.59* (1.07–2.37)		1.00 (0.89–1.11)
Educational attainment (years)	< 15		1.00 (reference)		1.00 (reference)
	10–12		1.04 (0.67–1.60)		0.81*** (0.74–0.90)
	≥ 19		1.31 (0.70–2.46)		0.69*** (0.61–0.78)
Household equivalised income	1st quintile (lowest)		1.00 (reference)		1.00 (reference)
	2nd quintile		0.71 (0.41–1.23)		0.95 (0.83–1.09)
	3rd quintile		1.13 (0.67–1.93)		0.76*** (0.67–0.86)
	4th quintile		0.83 (0.45–1.52)		0.67*** (0.57–0.79)
	5th quintile (highest)		0.85 (0.42–1.71)		0.66*** (0.57–0.76)
Cancer	No		1.00 (reference)		1.00 (reference)
	Yes		0.95 (0.32–2.81)		1.15 (0.93–1.43)

For peer review only

Heart disease	No	1.00 (reference)	1.00 (reference)
	Yes	1.16 (0.69–1.96)	1.20** (1.05–1.37)
Stroke	No	1.00 (reference)	1.00 (reference)
	Yes	1.28 (0.55–2.99)	1.18 (0.80–1.73)
Self-rated health	Good	1.00 (reference)	1.00 (reference)
	Poor	1.71* (1.13–2.58)	1.62*** (1.43–1.83)
CES-D score at baseline		1.89*** (1.59–2.25)	1.00 (reference)
GDS score at baseline			1.92*** (1.85–1.99)
Smoking	Never/past	1.00 (reference)	1.00 (reference)
	Current	0.74 (0.36–1.53)	1.29*** (1.13–1.48)
Drinking	Never/past	1.00 (reference)	1.00 (reference)
	Current	1.39 (0.80–2.40)	1.03 (0.93–1.14)

*, $p < 0.05$; **, $p < 0.01$; ***, $p < 0.001$; †, $p < 0.1$

ELSA, English Longitudinal Study of Ageing; JAGES, Japan Gerontological Evaluation Study; CES-D, Center for Epidemiologic Studies Depression Scale; CI, confidence interval; GDS, Geriatric Depression Scale; OR, odds ratio

For peer review only

STROBE Statement—Checklist of items that should be included in reports of ***cohort studies***

	Item No	Recommendation	Page No
**Title and abstract**	1	(*a*) Indicate the study's design with a commonly used term in the title or the abstract	1, 3
		(*b*) Provide in the abstract an informative and balanced summary of what was done and what was found	3-4
**Introduction**			
Background/rationale	2	Explain the scientific background and rationale for the investigation being reported	6-9
Objectives	3	State specific objectives, including any prespecified hypotheses	9
**Methods**			
Study design	4	Present key elements of study design early in the paper	9
Setting	5	Describe the setting, locations, and relevant dates, including periods of recruitment, exposure, follow-up, and data collection	9-10
Participants	6	(*a*) Give the eligibility criteria, and the sources and methods of selection of participants. Describe methods of follow-up	9-10
		(*b*) For matched studies, give matching criteria and number of exposed and unexposed	Not applicable
Variables	7	Clearly define all outcomes, exposures, predictors, potential confounders, and effect modifiers. Give diagnostic criteria, if applicable	10-11
Data sources/ measurement	8*	For each variable of interest, give sources of data and details of methods of assessment (measurement). Describe comparability of assessment methods if there is more than one group	10-11
Bias	9	Describe any efforts to address potential sources of bias	12
Study size	10	Explain how the study size was arrived at	Not applicable
Quantitative variables	11	Explain how quantitative variables were handled in the analyses. If applicable, describe which groupings were chosen and why	10-11
Statistical methods	12	(*a*) Describe all statistical methods, including those used to control for confounding	11-12
		(*b*) Describe any methods used to examine subgroups and interactions	Not applicable
		(*c*) Explain how missing data were addressed	12
		(*d*) If applicable, explain how loss to follow-up was addressed	Not applicable
		(*e*) Describe any sensitivity analyses	Not applicable
**Results**			
Participants	13*	(a) Report numbers of individuals at each stage of study—eg numbers potentially eligible, examined for eligibility, confirmed eligible, included in the study, completing follow-up, and analysed	13
		(b) Give reasons for non-participation at each stage	Not applicable
		(c) Consider use of a flow diagram	Not applicable
Descriptive data	14*	(a) Give characteristics of study participants (eg demographic, clinical, social) and information on exposures and potential confounders	13-14
		(b) Indicate number of participants with missing data for each variable of interest	15-16
		(c) Summarise follow-up time (eg, average and total amount)	10
Outcome data	15*	Report numbers of outcome events or summary measures over time	17-18

For peer review only

Main results	16	(*a*) Give unadjusted estimates and, if applicable, confounder-adjusted estimates and their precision (eg, 95% confidence interval). Make clear which confounders were adjusted for and why they were included	19-21
		(*b*) Report category boundaries when continuous variables were categorized	Not applicable
		(*c*) If relevant, consider translating estimates of relative risk into absolute risk for a meaningful time period	Not applicable
Other analyses	17	Report other analyses done—eg analyses of subgroups and interactions, and sensitivity analyses	Supplementary 1, 2
**Discussion**			
Key results	18	Summarise key results with reference to study objectives	23
Limitations	19	Discuss limitations of the study, taking into account sources of potential bias or imprecision. Discuss both direction and magnitude of any potential bias	26-28
Interpretation	20	Give a cautious overall interpretation of results considering objectives, limitations, multiplicity of analyses, results from similar studies, and other relevant evidence	23-26
Generalisability	21	Discuss the generalisability (external validity) of the study results	24-25
**Other information**			
Funding	22	Give the source of funding and the role of the funders for the present study and, if applicable, for the original study on which the present article is based	29-30

*Give information separately for exposed and unexposed groups.

Note: An Explanation and Elaboration article discusses each checklist item and gives methodological background and published examples of transparent reporting. The STROBE checklist is best used in conjunction with this article (freely available on the Web sites of PLoS Medicine at http://www.plosmedicine.org/, Annals of Internal Medicine at http://www.annals.org/, and Epidemiology at http://www.epidem.com/). Information on the STROBE Initiative is available at http://www.strobe-statement.org.

For peer review only
